# Diagnostic methods and therapeutic options of uveal melanoma with emphasis on MR imaging—Part I: MR imaging with pathologic correlation and technical considerations

**DOI:** 10.1186/s13244-021-01000-x

**Published:** 2021-06-03

**Authors:** Pietro Valerio Foti, Mario Travali, Renato Farina, Stefano Palmucci, Corrado Spatola, Luigi Raffaele, Vincenzo Salamone, Rosario Caltabiano, Giuseppe Broggi, Lidia Puzzo, Andrea Russo, Michele Reibaldi, Antonio Longo, Paolo Vigneri, Teresio Avitabile, Giovani Carlo Ettorre, Antonio Basile

**Affiliations:** 1 Department of Medical Surgical Sciences and Advanced Technologies “G.F. Ingrassia” – Radiology I Unit, University Hospital Policlinico “G. Rodolico-San Marco”, Via Santa Sofia 78, 95123 Catania, Italy; 2 Department of Medical Surgical Sciences and Advanced Technologies “G.F. Ingrassia” – Section of Anatomic Pathology, University of Catania, Via Santa Sofia 78, 95123 Catania, Italy; 3 Department of Ophthalmology, University of Catania, Via Santa Sofia 78, 95123 Catania, Italy; 4 Department of Clinical and Experimental Medicine, Center of Experimental Oncology and Hematology, University Hospital Policlinico “G. Rodolico-San Marco”, Via Santa Sofia 78, 95123 Catania, Italy

**Keywords:** Magnetic resonance imaging, Diffusion magnetic resonance imaging, Melanoma, Retinal detachment, Eye

## Abstract

Uveal melanoma is a malignant neoplasm that derives from pigmented melanocytes of the uvea and involves, in order of decreasing prevalence, the choroid, ciliary body and iris. Its prognosis is related to histopathologic and genetic features, tumor size and location, extraocular extension. The diagnosis is fundamentally based on clinical evaluation (ophthalmoscopy, biomicroscopy) and ultrasonography. MRI is useful in case of untransparent lens or subretinal effusion. Moreover, MRI has a significant role to confirm the diagnosis, in the evaluation of the local extent of the disease with implications for treatment planning, and in the follow-up after radiotherapy treatment. Uveal melanoma can show different morphologic features (lentiform, dome or mushroom shape) and often determines retinal detachment. MR appearance of uveal melanoma mainly depends on the melanin content. Uveal melanoma typically displays high signal intensity on T1-weighted images and low signal intensity on T2-weighted images. Nevertheless, imaging appearance may be variable based on the degree of pigmentation and the presence of areas of necrosis or cavitation. Differential diagnosis includes other uveal lesions. The radiologists and in particular MRI play a significant role in the clinical management of uveal melanoma. The purpose of this pictorial review is to provide the radiologists with awareness about diagnostic methods and therapeutic options of uveal melanoma. In the present first section we summarize the MR anatomy of the eye and describe ophthalmological and radiological imaging techniques to diagnose uveal melanomas, with emphasis on the role of MR imaging. Additionally, we review MR imaging appearance of uveal melanomas.

## Key points

- Uveal melanoma involves, in order of decreasing prevalence, choroid, ciliary body, iris.
- MRI has a role in characterization, treatment planning, follow-up of uveal melanoma.
- Typical uveal melanoma is hyperintense on T1-weighted and hypointense on T2-weighted images.
- Retinal detachment has a V-shape with the vertex on the optic disc.
- Retinal detachment does not enhance and does not show restricted diffusion, unless hemorrhagic.

## Introduction

Uveal melanoma is the commonest primary intraocular malignant neoplasm in adults and the second most common kind of melanoma [1]. It is relatively seldom; its incidence rate is of 5–7 cases per million person-years in Europe and 6 per million in the USA, with about 1500 new cases diagnosed each year. Its incidence has remained constant over last thirty years [1–3]. Epidemiological data and risk factors are summarized in Table 1 [4–6] and Table 2 [7–9], respectively.

**Table 1:** Epidemiological data of uveal melanoma

Parameter	Distribution
Incidence rate (million person/year)	5–7
Median age (range)	62 (6–100)
Gender (%)	
Male	51.2
Female	48.8
Race (%)	
Caucasian	98
Hispanic	1
American African	< 1
Asian	< 1
White-to-Black patients ratio	1:15–1:50
Uveal melanoma localization (%)	
Choroid	90
Ciliary body	7
Iris	3

**Table 2:** Risk factors and other worth mentioning features of uveal melanoma

Parameter	Distribution
Congenital risk factors	Light eye color, fair skin color, inability to tan and propensity to sunburn, oculodermal melanocytosis, cutaneous, iris and choroidal nevus, ancestry from northern latitudes
Main environmental risk factors	Welding, occupational cooking, sunlight exposure
Overall mortality rate	40–50% in 15 years
Metastases rate	50% of patients
Survival rate (month)	
With liver metastases	4–6
Other metastases	19–28
Metastatic location (%)	
Liver	89
Lung	29
Bone	17
Skin and subcutaneous tissue	12
Lymph nodes	11
Central nervous system	2
Predictive factors for metastatic disease	Basal tumor diameter, ciliary body involvement, extrascleral extension, epithelioid melanoma cytology, microvascular density

The neoplasm can involve any portion of the uveal tract (iris, ciliary body, choroid) [10]. Thorough awareness of the anatomy of the eye is mandatory since may facilitate the comprehension of the exact source of a pathology and therefore its characterization, narrowing the differential diagnosis. The diagnosis of uveal melanoma is based on ophthalmoscopy, biomicroscopy and ultrasonography (US). Magnetic resonance imaging (MRI) is useful in the event of untransparent lens or subretinal effusion. Furthermore, MRI has gained importance in treatment planning, in the evaluation of tumor response to radiotherapy and in the follow-up of patients with uveal melanoma, so much so that nowadays the radiologist is an integral part of the multidisciplinary decision-making process that characterize the clinical management of patients with uveal melanoma.

The prognosis is related to histopathologic features, tumor size and location, extraocular extension and genetic changes. In particular, chromosomal alterations and, above all, gene expression profile (Table 3) have shed light on the carcinogenesis of uveal melanoma and might have a clinical value as prognostic marker and in the detection of novel therapeutic targets in precision medicine [11–13].

**Table 3:** Chromosome aberrations and driver mutated genes in uveal melanoma and their prognostic implications

Genetic features	Clinical implications
Non-random chromosome aberrations	
Monosomy 3, gain of chromosome 8q	High metastatic risk
Gain of chromosome 6p	Low metastatic risk
Driver genes (mutations)	
BAP1	High metastatic risk, poor survival
EIF1AX	Increased patient survival
SF3B1	Late-onset metastatic disease
GNA11, GNAQ	No prognostic value, not linked to patient outcomes

Although the finding of distant metastasis is seldom at the time of initial diagnosis (< 5% of patients) [3], about 50% of patients with uveal melanomas develop metastasis during the course of the disease [14, 15], metastatization still representing the major cause of death [1]. Metastatic spread of uveal melanomas is hematogenous, with a predilection for the liver (89%), followed by lung (29%), bone (17%), skin (12%) and lymph nodes (11%); also the brain can be involved with a lower rate [8]. The 10-year metastatic rate varies depending on tumor location as follows: ciliary body 33%, choroid 25% and iris 7% [16].

Therapy of uveal melanoma aims to preserve the eye and its function and to avoid, wherever possible, metastatic dissemination. In the last decades globe-conserving therapies have largely replaced surgical procedures, nevertheless treatment selection is very complex and must keep into account many variables.

This pictorial essay aims to provide the radiologists with awareness about diagnostic methods and therapeutic options of uveal melanoma. In the present first instalment we expose the epidemiological, clinical and histopathologic features of uveal melanomas. We summarize the anatomy and MR anatomy of the eye, the most frequent technical issues encountered when imaging the orbit as well as their possible solutions. We describe ophthalmological and radiological imaging techniques to diagnose uveal melanomas, with emphasis on the role of MR imaging. We review MR imaging semeiotics of uveal melanoma (in its possible variants) with pathologic radiologic correlations, using examples from our institution; the main differential diagnoses are also considered.

In the following second section we will present the therapeutic management of uveal melanoma, illustrating the main globe-retaining therapies, their indications and complications.

## Clinical features

Clinical symptoms of uveal melanoma are related to the location and size of the neoplasm and may vary from asymptomatic (lesion accidentally detected at a routine ophthalmological examination, about 30% of cases) to complete visual loss. The most frequent symptoms include blurred vision, defects of the visual field, photopsia (flickering or flashing of light), metamorphopsia (distorted vision), irritation, pain, floaters, redness, increased intraocular pressure.

**Fig. 1:**
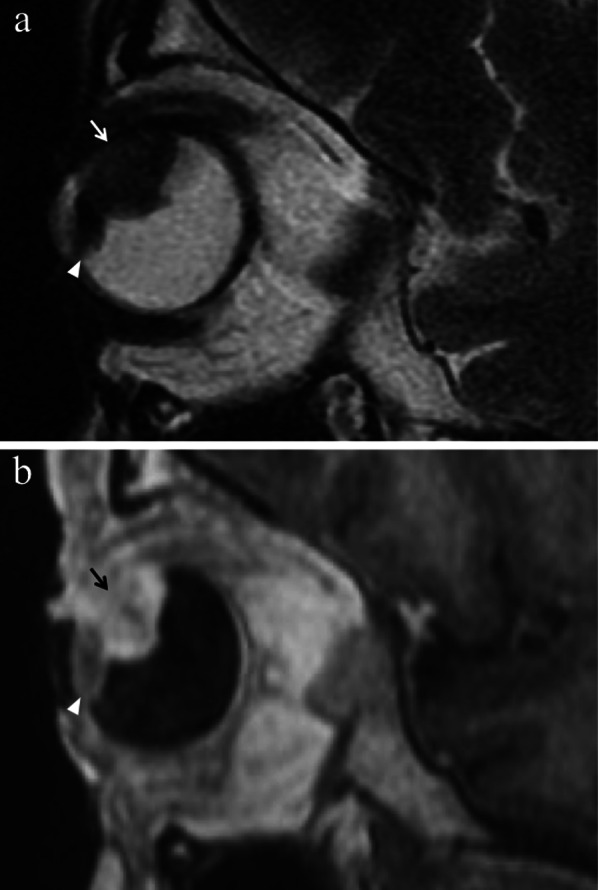
A 40-year-old male welder with a ciliary body melanoma of the left eye determining lens displacement. Sagittal (**a**) T2-weighted turbo spin-echo STIR and (**b**) contrast-enhanced 3D T1-weighted images show a mass (arrows) along the antero-superior aspect of the globe, displacing the lens (white arrowheads). The lesion is hypointense on T2-weighted image (white arrow) and demonstrates enhancement on post-contrast T1-weighted image (black arrow)

Location-related symptoms can be as follows:

- iris melanoma: growth of preexisting iris lesion, novel pigmented lesion of the iris (80% of cases in the inferior half), corectopia (pupil distortion), heterochromia (iris color variation), hyphema (collection of blood into the anterior chamber), localized cataract, secondary glaucoma, episcleral injection, chronic conjunctivitis, ectropion uveae;
- ciliary body melanoma: lens displacement with refractive disorders (i.e., asymmetric astigmatism) (Fig. 1), cataract, increased intraocular pressure [1, 3, 4].

Generally, at the time of diagnosis iris melanoma is smaller than ciliary body melanoma due to the visible location of the former [5]; for the same reason, iris melanoma is commonly diagnosed 10–20 years earlier than that of ciliary body or choroid [4].

## Pathologic features

Three histologic types of uveal melanoma are identified:

1. S*pindle shaped melanoma,* whom cells have a high nuclear to cytoplasmic ratio, oval nuclei with clumped chromatin and prominent nucleoli. Few mitoses are generally observed. This cell type is associated with the best prognosis.
2. *Epithelioid cell melanoma* composed of large cells with acidophilic glassy cytoplasm and large, round, vesicular nuclei with prominent nucleoli. This cell type is associated with a high risk of metastasis.
3. *Mixed melanoma*, composed by variable numbers of both cell types, has an intermediate prognosis. Tumors with 3–5% of epithelioid cells are considered mixed.

**Fig. 2:**
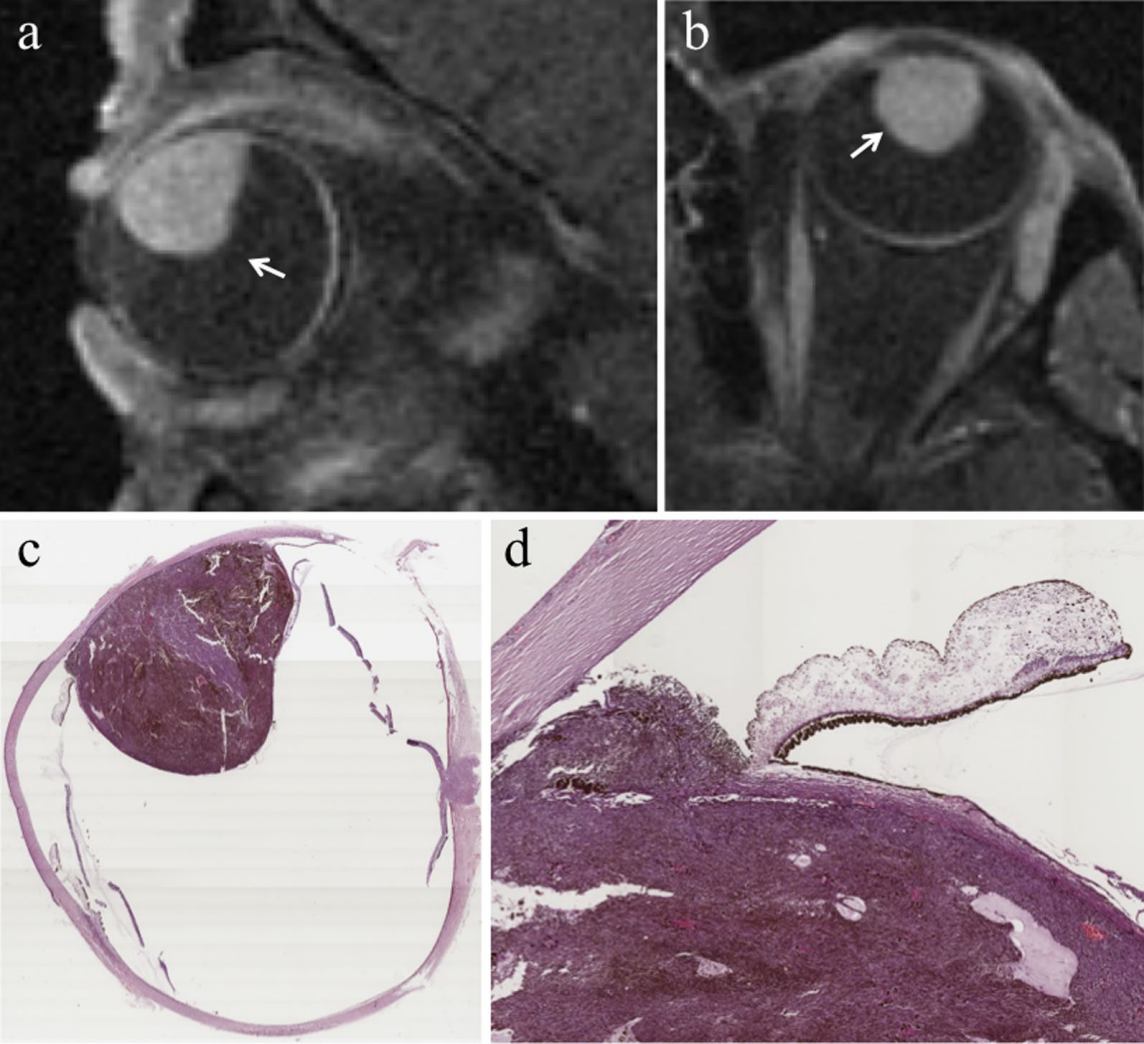
A 40-year-old man with a ciliary body melanoma of the left eye. The same patient as in Fig. 1. (**a**) Sagittal and (**b**) axial contrast-enhanced fat-suppressed T1-weighted images illustrate a homogeneously enhancing intraocular lesion along the antero-superior aspect of the globe (white arrows), with ciliary body invasion. **c** On low magnification histological examination shows a strong overlap with MR imaging: a pigmented mass that protrudes into the posterior segment of the eye is clearly identifiable (H&E, original magnification ×25). (**d**) Higher magnification showing the initial invasion of the ciliary body by melanoma cells (H&E, original magnification ×50)

Lymphocytic infiltration in uveal melanoma is not observed as frequently as in cutaneous melanoma. The two types of lymphocytic infiltrates are patchy and diffuse. The most important negative prognostic factors are tumor size, ciliary body involvement (Fig. 2), extraocular extension, monosomy of chromosome 3 and loss of nuclear BAP1 expression [17].

It is important to know that uveal melanomas in contact with aqueous humor may incur cytological modification exhibiting downgrading of atypia and decrease of proliferation indices [18]. This event concerns primary iris melanomas and ciliary body melanoma invading the iris surface and is thought to be due to the antineoplastic activity of ascorbic acid, whose concentration is 15–20-fold higher in the aqueous humor than in the plasma [19, 20]. Pathologists should take into account this phenomenon when interpreting suspected iris tumor biopsy [21].

## Anatomical notes and MR anatomy of the eye

**Fig. 3:**
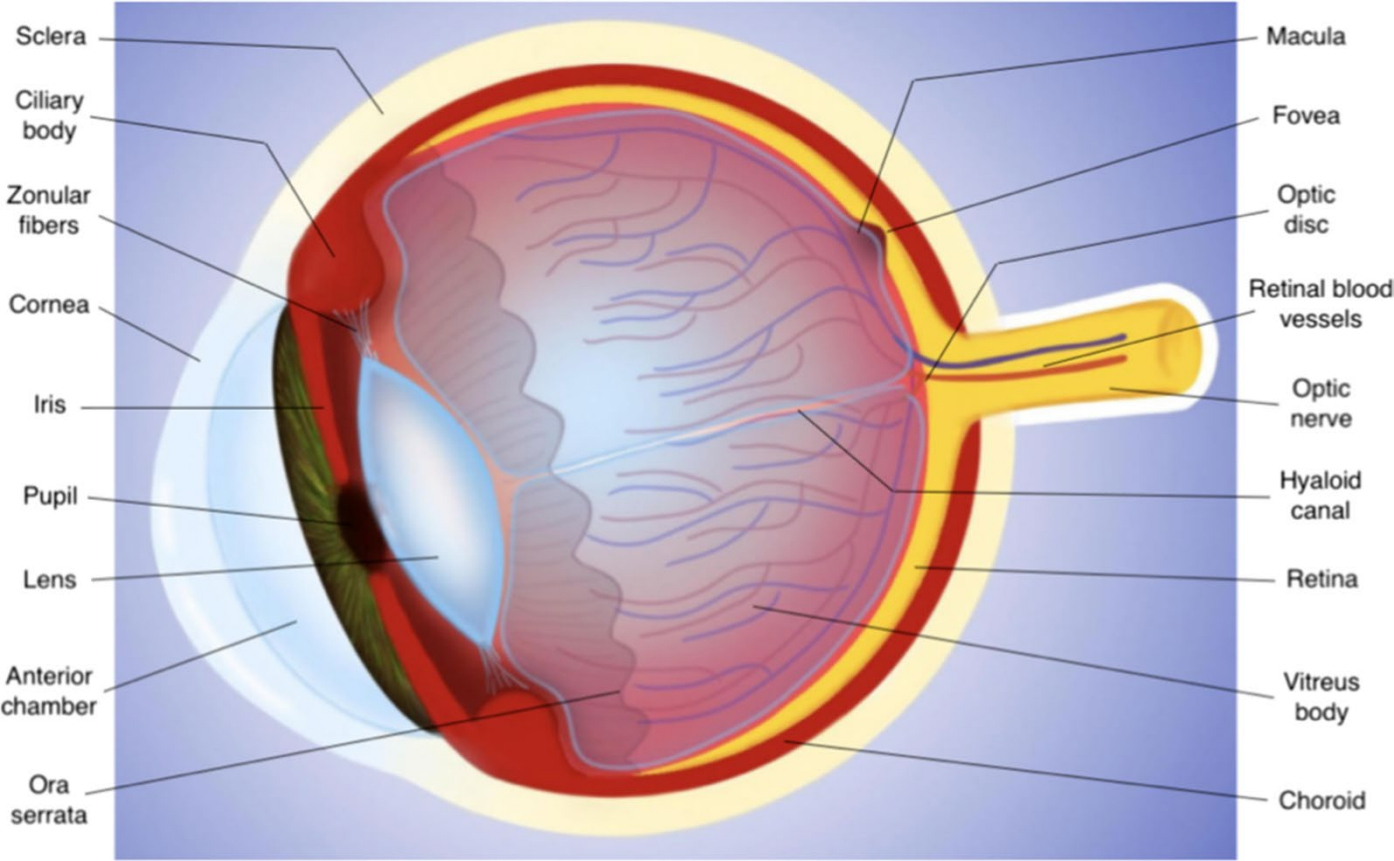
Drawing of the eye anatomy in the midsagittal section

**Fig. 4:**
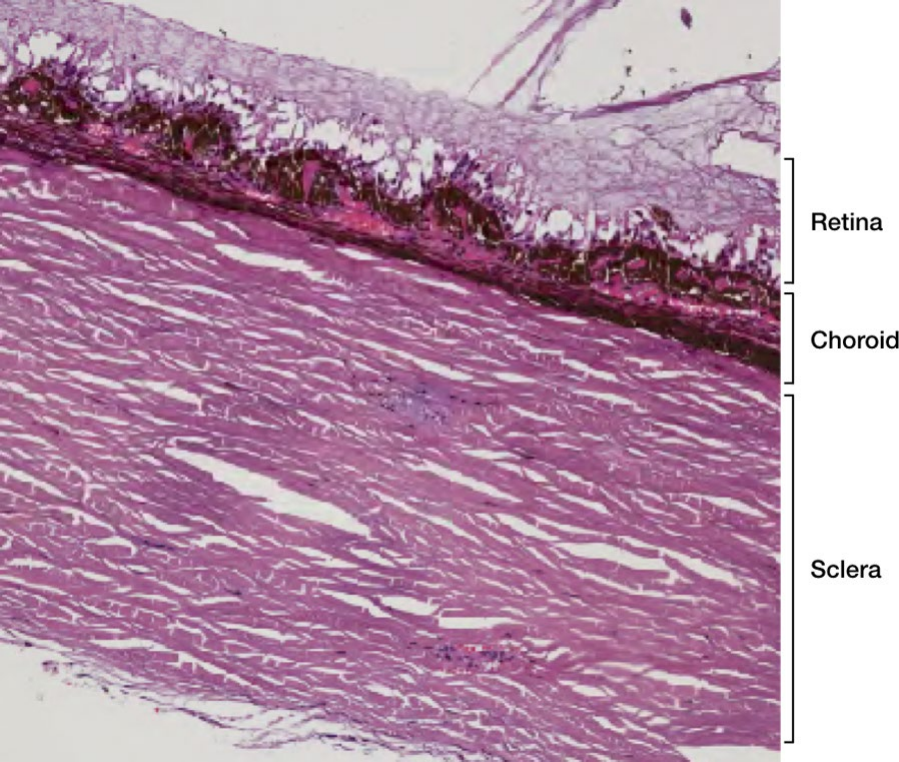
Histological detail illustrating a full-thickness section of the posterior wall of the eye, consisting of three layers (from outer to inner): sclera, choroid and retina (H&E, original magnification ×200)

The wall of the globe is composed of three main layers (Figs. 3 and 4):

- the *sclera*, the outer fibrous avascular coat, with the transparent cornea anteriorly;
- the *uvea*, the middle vascular pigmented tunic, comprising, from forward to backward, the iris, ciliary body and choroid;
- the *retina*, the inner neural, sensory layer, merging posteriorly with the optic nerve [22].

**Fig. 5:**
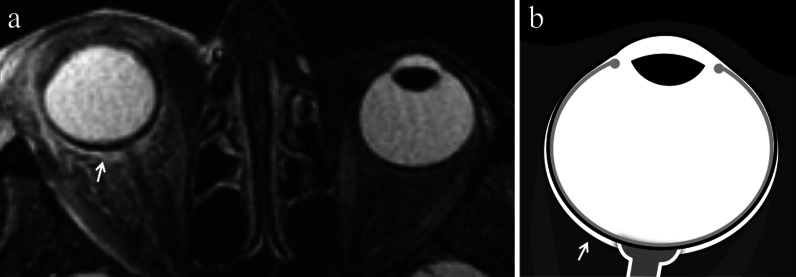
A 91-year-old woman with orbital cellulitis and endophthalmitis of the right eye. (**a**) Axial T2-weighted turbo spin-echo STIR image displays a fluid collection, adjacent to the posterior outer edge of the sclera of the right eye, filling the Tenon’s space (white arrow). Note the edematous thickening of the periocular tissues and exophthalmos. (**b**) Corresponding schematic drawing illustrates the anatomic location of the episcleral (Tenon’s) space (white arrow)

A fascial fibroelastic sheath, referred to as *Tenon’s capsule*, envelops the eyeball. Between the sclera and Tenon’s capsule there is a virtual recess, the *episcleral (Tenon’s) space*, in which pathologic processes can seep (Fig. 5) [22].

The uvea arises from the mesenchyme and takes its name from the Latin “*uva”* or grape [23]. At the end of the first month of gestation, melanocyte precursors, originating from the neural crest, migrate inside the uvea and then differentiate as *pigmented melanocytes*; uveal melanomas derive from these melanocytes. Histologically, the uvea is made up of a stroma of collagenous and elastic connective tissue that supports blood vessels, immune cells (mast cells, macrophages, lymphocytes) and melanocytes; these latter are closely apposed to the blood vessels. The primary role of the uvea is to supply oxygen and nutrients to the outer layer of the retina; other functions encompass light sorption, thermoregulation, intraocular pressure regulation. On the other hand, the role of the melanocytes is still not exactly known [24].

The *uveal tract* encompasses the iris, ciliary body and choroid. The *iris* is a thin muscular diaphragm regulating the pupil diameter. The outer aspect of the iris attaches to the *ciliary body*. The latter constitutes a complete muscular ring running around the inner aspect of anterior sclera. It is connected to the lens through the zonular fibers or suspensory ligaments and alters the curvature of the lens during accommodation. Posteriorly the ciliary body merges with the choroid at the ora serrata. The *choroid* extends from the ora serrata to the optic nerve head; it is the most vascularized portion of the eye. Its inner surface is attached to the retina, whereas the outer surface is attached to the sclera. In particular, the *Bruch’s membrane* is the innermost layer of the choroid, in contact with the retinal pigment epithelium (RPE). The binding between the choroid and sclera is stronger where the posterior ciliary arteries and ciliary nerve join the globe and where the vortex veins leave the eyeball. This vascular anatomy explains the typical biconvex shape of choroidal detachment due to tethering at the level of the vascular poles (cf. section differential diagnosis) [22].

When it comes to ocular detachments, it is important to remember two virtual spaces of the eye in which fluids may collect: (a) the *subretinal spa*ce, virtual space between the sensory retina and the RPE, anatomical site of retinal detachments; (b) the *suprachoroidal space*, virtual space between the choroid and sclera, anatomical location of choroidal detachments [23].

### MR anatomy

**Fig. 6:**
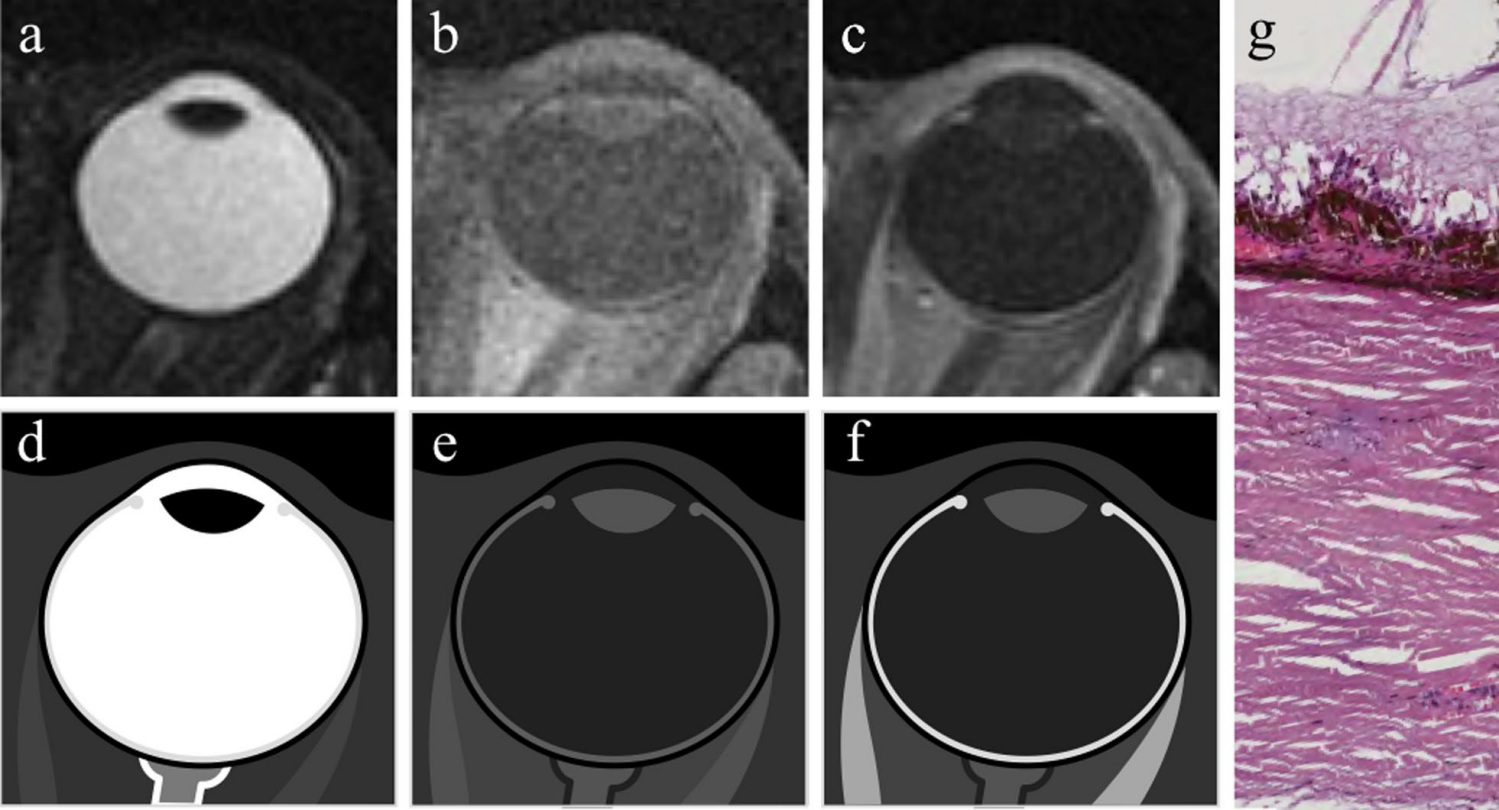
MR anatomy of the eye. Axial (**a**) T2-weighted turbo spin-echo STIR, (**b**) fat-suppressed T1-weighted, (**c**) contrast-enhanced fat-suppressed T1-weighted images. **d–f** Corresponding schematic drawings. (**g**) Corresponding histological detail illustrating the three-layered wall of the globe: (from outer to inner) sclera, choroid and retina (H&E, original magnification ×200)

The *cornea* has a thickness of 0.5 mm. At MRI it shows low signal intensity because of collagen content. The *sclera* (thickness 0.3–1 mm) is composed of collagen too, and exhibits low signal intensity on all pulse sequences (Fig. 6).

The *uveal tract* (thickness 0.1–0.22 mm at the level of the choroid) displays slightly high signal intensity on T1-weighted images and low signal intensity on T2-weighted images, although on this latter sequence it is partially obscured by the high signal of aqueous and vitreous humor.

The thickness of the *retina* ranges from 0.056 mm and 0.1 mm [23]. Under physiological conditions, the retina is closely connected to the choroid and cannot be perceived individually.

After contrast media administration the uveal tract and retina demonstrated enhancement on T1-weighted images. Therefore, differently from T1-weighted images, on T2-weighted sequences the different layers cannot be differentiated and the wall of the eye appears as an evenly hypointense line.

When retinal or choroidal detachments occur, then the different layers of the eyeball can be separately detected.

The *lens* shows a typical biconvex shape and, due to its high protein content, displays intermediate signal intensity on T1-weighted images and low signal intensity on T2-weighted images.

The *aqueous humour* and *vitreous body* have a water content greater than 98% and therefore exhibit a water-like signal intensity at MRI, appearing dark and bright on T1- and T2-weighted images, respectively [22, 23, 25, 26].

According to Ferreira et al. [26] the *Tenon’s space* would be visible as a thin layer, slightly hyperintense on T1- and T2-weighted images, just outside the globe.

## Imaging methods in the diagnosis of uveal melanoma

### Clinical evaluation

The diagnosis of uveal melanoma is fundamentally based on clinical evaluation by means of *slit-lamp biomicroscopy* for the assessment of the anterior segment (iris melanoma), and *indirect ophthalmoscopy* for the study of the posterior segment of the eye (ciliary body and above all choroidal melanoma) [4, 14]. Clinical diagnosis of uveal melanoma has an accuracy of 99.7% when conducted in reference centers [27]. Through these techniques the ophthalmologist is able to assess various morphologic features of the lesion such as: location, shape, pigmentation, distance from the optic nerve head, invasion of the ciliary body [14]. However, in order to confirm the diagnosis, a variety of imaging methods can be employed as well.

### Ophthalmological imaging techniques

#### Gonioscopy

Gonioscopy is a diagnostic method based on gonioscopic lens that allows to study the peripheral portion of the anterior segment. It enables to evaluate the invasion of the anterior chamber angle by a melanoma of the iris or ciliary body [14].

#### Transillumination

During transillumination a bright light is projected into the conjunctival fornix opposite the melanoma and produces a shadow through the sclera outlining the lesion. It is used to verify the involvement of the ciliary body [14].

#### Ultrasonography (US) and ultrasound biomicroscopy (UBM)

Ocular US is an essential imaging tool both in the diagnosis and in the follow-up of posterior uveal melanomas undergoing conservative treatment. US is particularly useful in the event of opaque media (i.e., cataract and hemorrhage of the vitreous), when the tumor cannot be clinically visualized. Ocular US (frequency 10 MHz) can be performed with two different methods: A-scan and B-scan.

At A-scan US uveal melanoma demonstrates medium to low internal reflectivity decreasing toward the sclera. B-scan US is useful to assess morphologic features of the tumor such as extension, shape (flat-, dome- or mushroom-shaped), size, and possible extrascleral extension. B-scan US may also detect spontaneous vascular pulsations, expression of a highly vascularized solid neoplasm and useful in differentiating this latter from hemorrhage.

High-frequency (12.5–100 MHz) UBM is an imaging tool providing high-resolution images of the anterior segment, since the high-frequency probe manages to penetrate up to 4–5 mm in depth. It is suitable to study melanomas of the iris and ciliary body, to detect whether an iris lesion has invaded the ciliary body and to search for extrascleral extension [4, 14, 28–30].

#### Fundus fluorescein angiography (FFA)

FFA evaluates retinal and choroidal circulation as well as intrinsic tumor vasculature and is useful in the differential diagnosis of choroidal lesions demonstrating different vascular pattern. It can highlight retinal pigment epithelium abnormalities related to the tumor. Moreover, FFA is suitable to assess complication following brachytherapy (radiation retinopathy and maculopathy) [14, 28].

#### Optical coherence tomography (OCT)

OCT is an imaging technique capable of studying the posterior segment. It has a role in diagnosis, treatment planning and follow-up of neoplastic lesions. OCT allows to evaluate the posterior vitreous, retina, choroid and sclera and is particularly useful to image small melanomas, less than 3 mm thick. Moreover, OCT angiography has an important indication in detecting radiotherapy-related macular microangiopathy [14, 28].

#### Fine-needle aspiration biopsy (FNAB)

Due to high accuracy of clinical diagnosis and the risk of procedure-related complications (i.e., vitreous hemorrhage), biopsy of the lesion is not recommended for uveal melanoma; however, FNAB may be conducted, through both a trans-scleral or trans-vitreal approach, in those rare cases in which uncertainty about the clinical diagnosis subsists or, more seldom, for prognostic purposes [27].

In some centers tumor biopsy is performed after radiotherapy in order to avoid the, albeit rare, risk of subconjunctival neoplastic seeding [31].

### Radiologic imaging techniques

#### Computed tomography

Although seldom used to study uveal melanoma, CT can be employed to assess particularly large tumors with orbital invasion [14]. At CT uveal melanomas are detectable as hyperdense lesions demonstrating enhancement after i.v. administration of iodinated contrast agents [32].

#### Positron emission tomography/computed tomography (PET/CT)

PET/CT does not offer a superior contribute, compared to that of clinical examination and cross-sectional imaging methods (CT and MRI), in detecting primary ocular melanoma. On the other hand, since it is a comprehensive technique capable of allowing a whole body assessment, PET/CT is useful to identify metastases from uveal melanoma and to assess response to treatment [33]. PET/CT is superior to CT in characterizing bone lesions as benign or malignant [33] and may also have a role in assessing the metastatic potential of uveal melanoma, since the standardized uptake value (SUV) positively correlates with largest tumor diameter and cell type (epitheloid) [34].

#### Magnetic Resonance Imaging – Role of MRI

The role of MR imaging in uveal melanoma is manifold:

- detection,
- characterization (origin of the tumor),
- evaluation of the local extent of the disease – treatment choice and planning,
- assessment and prediction of tumor response to radiotherapy,
- follow-up and evaluation of treatment-related complications.

MRI is particularly useful to evaluate uveal melanoma in eyes in which opaque media (cataract, hemorrhage of the vitreous) hamper the use of indirect ophthalmoscopy in the study of the posterior segment of the eye. Other important contributions provided by MR are the measurement of the basal diameters and maximum prominence (tumor thickness including sclera thickness) of uveal melanoma (especially of the ciliary body) and the evaluation of extrascleral and orbital extension of large uveal melanomas [14, 26, 35].

A thorough assessment of the dimensions and the geometry of the tumor is mandatory for treatment planning; in this setting, pretreatment imaging plays a pivotal role when choosing the most suitable therapeutic strategy.

Routinely, uveal melanomas dimensions are evaluated through US with high frequency probes. Nonetheless, due to the often irregular shape of the lesion, 2D US measurements may be operator-dependent. In particular, the anatomy of the splanchnocranium (i.e., the nose) may prevent the proper orientation of the US probe, thus leading to oblique measurements that overestimate the tumor diameter. Besides, in case of large tumors the basal diameter may be too large for the limited field of view of the US transducer. Not least, in some patients previously treated for retinal detachment, silicone oil is employed as a tamponade; this makes particularly challenging or even impossible US examination, since silicon oil–water interface reflects the sound waves [10].

MRI bears numerous advantages as compared with US. Its intrinsic multiparametric nature allows for excellent contrast resolution and multiple tissue-contrasts to be gained. The considerable contrast between the sclera and periocular adipose tissue allows for a better definition of tumor margins. Moreover, the 3D volumetric nature of the data acquired by MRI offers an optimal definition of the geometrical properties of the lesion that makes the measurement of the tumor diameters more accurate, even in case of large lesions consisting of various lobes. In particular, the isotropic spatial resolution of 3D acquisitions enables MR sequences to be reformatted into high-resolution images with countless orientations [10, 35]. Additionally, using a properly modified MR protocol it is possible to image patients previously treated for retinal detachment with silicon oil, the eyes of whom in the past needed to be enucleated because of the impossibility to perform a correct follow-up [36]. For these reasons, the inclusion of an MR examination in the diagnostic management of selected patients with uveal melanoma may positively impact the therapeutic planning, thus changing treatment procedure from enucleation to proton beam therapy or to the less expensive plaque brachytherapy. This improves the quality of care, enhances the possibility to spare vision and reduces the economic burden [10].

Quantitative diffusion‑weighted MR imaging (DWI) demonstrated to be a valuable tool in noninvasively predicting and detecting the response of uveal melanomas to radiotherapy. In particular, during the follow-up of patients undergoing proton beam therapy, a significant increase of apparent diffusion coefficient (ADC) precedes the macroscopic changes in tumor volume. Moreover, the pretreatment ADC value significantly correlates with tumor regression. (The lower the ADC, the better is the treatment response.) These findings are particularly relevant since expand the role of quantitative MR imaging biomarkers from diagnostic to prognostic and could guide a more personalized therapy, thus improving patients’ care [26, 37–39].

The *MR imaging protocol* employed to study uveal melanoma must encompass sequences with both anatomical and functional purposes, thus exploiting the multiparametric capabilities of MRI. The standard MR protocol includes high-resolution T1- and T2-weighted sequences with or without fat suppression, DWI sequences and fat-suppressed T1-weighted sequences after i.v. administration of paramagnetic contrast agent. Some authors also include a dynamic contrast-enhanced (DCE) sequence [26].

*High-resolution 2D sequences (T1-weighted and T2-weighted)* are particularly useful to evaluate eye anatomy (globe layers), lesion margins and shape, ciliary body and scleral infiltration, extrascleral extension. In particular, to identify the layer of origin of the mass is relevant for the differential diagnosis. On the other hand, 2D sequences are not suitable for multiplanar reformations; therefore, they should be acquired perpendicular or parallel to the long axis of the lesion [26].

*3D isotropic sequences* simultaneously acquire a complete volume and allow to obtain high-quality multiplanar reformations in all spatial planes; for this reason, they can be acquired in the axial plane, without a specific orientation. Owing to their multiplanar capabilities, 3D sequences are useful to evaluate lesion geometry and to perform thorough measurements; nevertheless, they are capable to evaluate scleral/extrascleral invasion as well. 3D sequences can be acquired with both gradient-echo and spin-echo fashion; the former have superior contrast resolution, but are more prone to susceptibility artifacts than spin-echo sequences, the latter better identify the outer scleral border [26].

When imaging the orbit, among the methods to achieve fat suppression, *STIR* sequences are more efficient to suppress the signal from fat than *frequency-selective fat saturation* sequences. In *STIR* sequences the suppression of the fat signal is T1 specific, namely relies on differences in the T1 of the various tissues, and is achieved by applying an inversion-recovery pulse. *STIR* sequences are not influenced by magnetic field inhomogeneity and allow to obtain a homogeneous and global fat suppression. On the other hand, in *frequency-selective fat saturation* sequences the suppression of the signal from fat is tissue specific, based on intrinsic differences in resonance frequencies between lipid protons and water protons. On *frequency-selective fat saturation* sequences incomplete fat saturation often occurs due to incorrect selective excitation of the fat resonance frequency caused by magnetic field inhomogeneities related to local magnetic susceptibility differences occurring at air–bone interfaces [40, 41]. However, after Gd-based contrast agent administration, *frequency-selective fat saturation* sequences are preferable to *STIR* sequences, since tissues with high accumulation of contrast agent may acquire a T1 similar to that of fat; accordingly, their signal may be suppressed on *STIR* images [41].

*High-b-value DWI* allows for evaluation of tumor cellularity and microstructure. DW sequences are useful to differentiate between benign and malignant lesions, to distinguish uveal melanoma from retinal detachment, to early detect and even predict uveal melanoma response to radiotherapy noninvasively [26, 37–39]. When it comes to DWI, the non-echo planar imaging (EPI) technique (i.e., turbo spin-echo TSE) is more suitable than the generally used EPI one, inasmuch the former is less prone to magnetic field inhomogeneity. Moreover, considering the limited field of view (FOV) and the reduced thickness of MR sequences employed in the study of the orbit (all factors that decrease the signal-to-noise ratio (SNR)), it would be more appropriate to acquire DWI sequences with a *b* value of 800 s/mm^2^ rather than a *b* value of 1000 s/mm^2^ in order to enhance the SNR [26].

*Dynamic contrast-enhanced magnetic resonance imaging (DCE-MRI)* is a functional MR technique which enables to evaluate in vivo the perfusion of a lesion. DCE-MRI implies the sequential (multiphasic) acquisition of multiple T1-weighted sequences (total of 50–95 temporal points) with high temporal resolution (acquisition time 3–9 s), before (5–9 baseline dynamic acquisitions), during and after i.v. administration of Gd-based contrast agents [26, 42, 43]. The tumor perfusion can be graphically represented in the shape of time-intensity curves (TIC) obtained by drawing a region of interest (ROI) in the lesion. Quantitative pharmacokinetic parameters, mirroring tumor microcirculation, can be obtained as well using dedicated postprocessing tools. The acquired images may, however, be susceptible to misregistration artifacts related to eye motion [26, 42, 43]. DCE-MRI in association with DWI can be useful to distinguish benign from malignant orbital lesions [43]. Furthermore, DCE sequences could have a prognostic relevance in distinguishing uveal melanomas with metastatic and nonmetastatic potential [42].

#### Technical issues in MR imaging of the orbit

Imaging the orbit implies a variety of technical issues to face with. These concern the choice of the coil, magnetic susceptibility effects and motion artifacts.

Dedicated surface receive coils, closely applied to the globe, allow for selective ocular imaging and are able to enhance both the SNR and spatial resolution (smaller pixel size at a given matrix). The resulting increased SNR may allow for a shorter scanning time [10]. On the other hand, because of their limited field of view (3–6 cm), surface coils do not allow the assessment of the deeper orbit and of the visual pathway extending beyond the orbital apex, including the canalicular portion of the optic nerve, optic chiasm, optic tracts, optic radiations and occipital cortex. Other drawbacks of surface coils encompass major sensitivity to chemical shift artifacts and magnetic susceptibility artifact at interfaces [22, 26]. In addition, dedicated specialized commercial radiofrequency coils are available only at few facilities. In these centers, a variety of dedicated receive single loop “micro-coils” of various sizes, in association with the body-transmit coil, can be employed at both 3- and 7-T MRI scanners [10, 44].

The anatomical location of the eye, adjacent to the air-tissue and bone-tissue interfaces, generates important magnetic susceptibility effects translating into noticeable geometric distortion and signal dropouts. This kind of artifact is stronger on gradient-echo than on spin-echo sequences [26]. A way to reduce susceptibility artifacts on the anterior side of the eye is to place a wet gauze on the closed eyelids of the affected eye [10, 35]. Obviously, before MR examination is mandatory to remove eye makeup, containing metallic pigments that may undermine image quality [22].

Voluntary and involuntary movements of the eyes can be detrimental for MR images. Various attempts have been made to reduce eye movement and enhance the quality of MR images. In the late 1990s, retrobulbar anesthesia has been proposed to suppress motion artifacts in patients with uveal melanoma. Although it is a valuable technique to obtain high-quality ocular MR images in cases of inconclusive ultrasonographic examinations, retrobulbar anesthesia is an invasive procedure not exempt of potential complications (retrobulbar hemorrhage, globe perforation, intraocular hemorrhage, retinal detachment) and must be conducted by an experienced ophthalmologist [45]. A method to minimize motion artifacts can be to image the eye closed with contralateral fixation of gaze, inasmuch closing the eyelids on one side cuts down involuntary movements and, at the meanwhile, contralateral fixation of gaze reduces voluntary movement of both eye due to conjugate gaze [46]. In order to reduce motion and blink-induced artifacts, some authors developed a “cued-blinking paradigm” in which the patient is invited to stare at a fixation target into the magnet with the unaffected eye and to blink at precise intervals [10]. Other authors just use to invite patients to keep their eyes closed and to reduce eye movement at a minimum during scanning [26].

##### Ultra-high-field 7 T MRI

The main potential advantage of 7 T MRI is the increased spatial resolution in comparison with that achievable at 1.5 T and 3 T. Nevertheless, some challenging issues must be kept into account with ultra-high-field MR scanners. First, the major sensitivity to magnetic susceptibility effects may potentially result in image distortion. Second, the rise in tissue T1 relaxation times and reduction in T2 relaxation times and T1-tissue contrast imply a re-optimization of scanning protocols [35]. Furthermore, it is worth recalling that specific absorption rate (SAR) increases with the square of the magnetic field strength. Accordingly, more heat is transferred into the globe at 3 T then at 1.5 T; in particular, this can become a crucial issue when imaging at 7 T [22].

## MR imaging with pathologic correlation

**Fig. 7:**
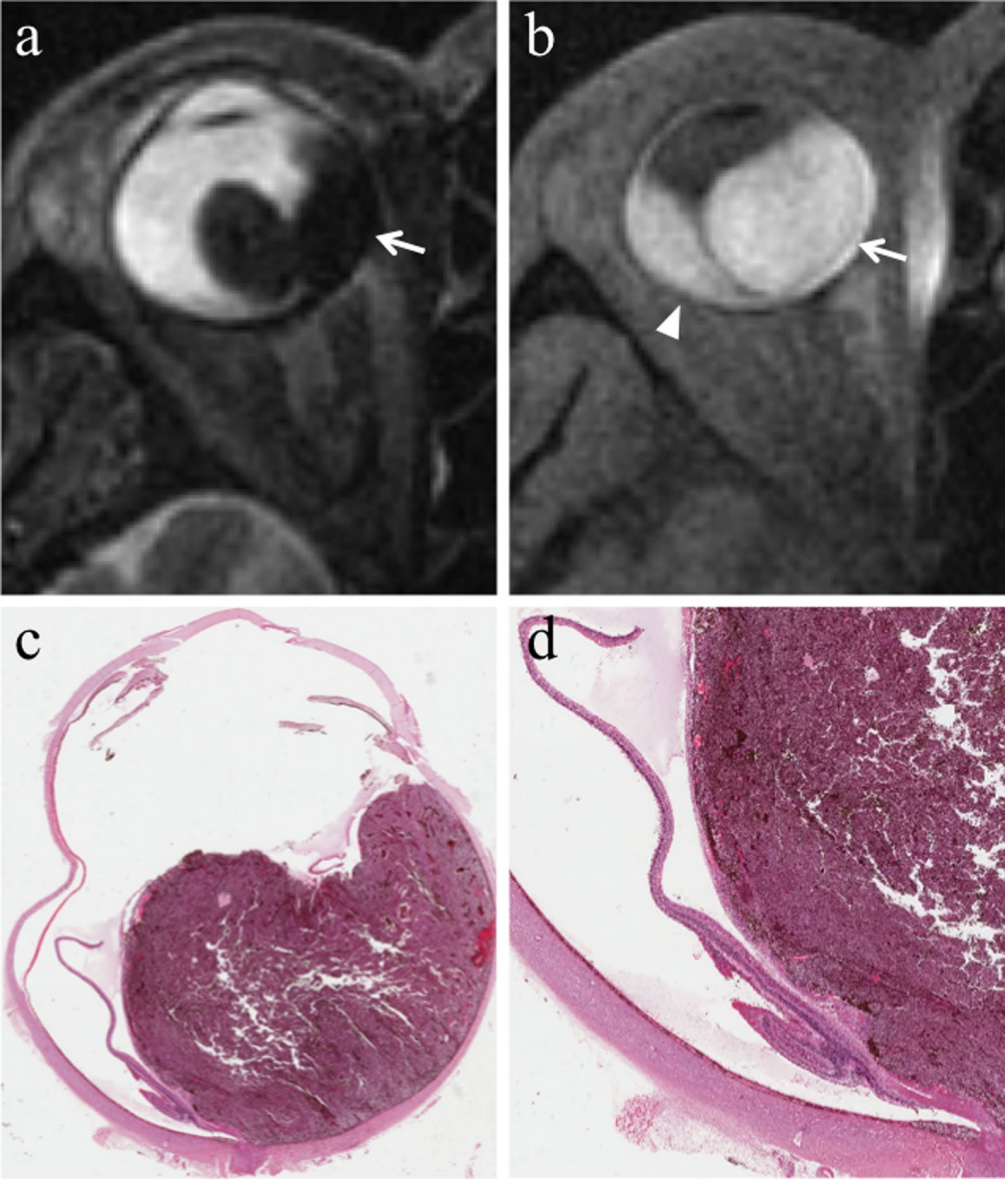
A 77-year-old man with a pigmented choroidal melanoma and hemorrhagic retinal detachment of the right eye. Axial (**a**) T2-weighted turbo spin-echo STIR and (**b**)( fat-suppressed T1-weighted images demonstrate an intraocular mushroom-shaped lesion along the postero-medial aspect of globe (white arrows). The mass exhibits the typical low signal intensity (lower signal intensity than vitreous body) on T2-weighted image and high signal intensity (higher signal intensity than vitreous body) on T1-weighted image. Along the postero-lateral aspect of globe, the hemorrhagic retinal detachment is better depicted on fat-suppressed T1-weighted image in which it displays high signal intensity due to subacute blood products (white arrowhead in **b**). (**c**) Histological examination: low magnification confirming the radiological finding of an intensely pigmented mass protruding from the postero-medial ocular wall (H&E, original magnification ×25). (**d**) The postero-lateral retinal detachment is also well documented at histological level (H&E, original magnification ×50)

Since uveal melanomas originate from melanocytes of the uvea, the MR appearance of uveal melanomas largely depends on the melanin content, varying from highly pigmented to amelanotic [47]. MR appearance of the different variants of uveal melanoma, as well as retinal and choroidal detachment, is described in Table 4. Melanin has a paramagnetic effect with T1 and T2 shortening; therefore, uveal melanoma typically displays high signal intensity (higher signal intensity than vitreous body) on T1-weighted images and low signal intensity (lower signal intensity than vitreous body) on T2-weighted images (Fig. 7). This finding is an important diagnostic clue in the differential diagnosis, inasmuch uveal melanoma is the sole ocular neoplasm demonstrating such an MR imaging semiotics (with very few exceptions) [47, 48].

**Table 4:**
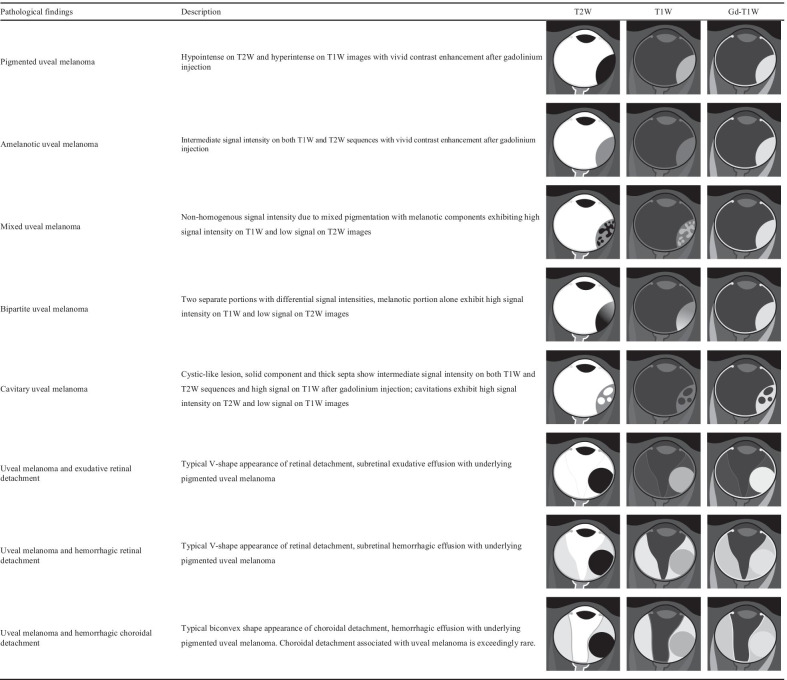
Chart summarizing MR imaging features of the different variants of uveal melanoma, retinal and choroidal detachment

According to some authors [49], the hyperintensity of melanin on T1-weighted images should be due to the binding of paramagnetic metals, related to its cytoprotective role as intracellular scavenger of metal ions.

**Fig. 8:**
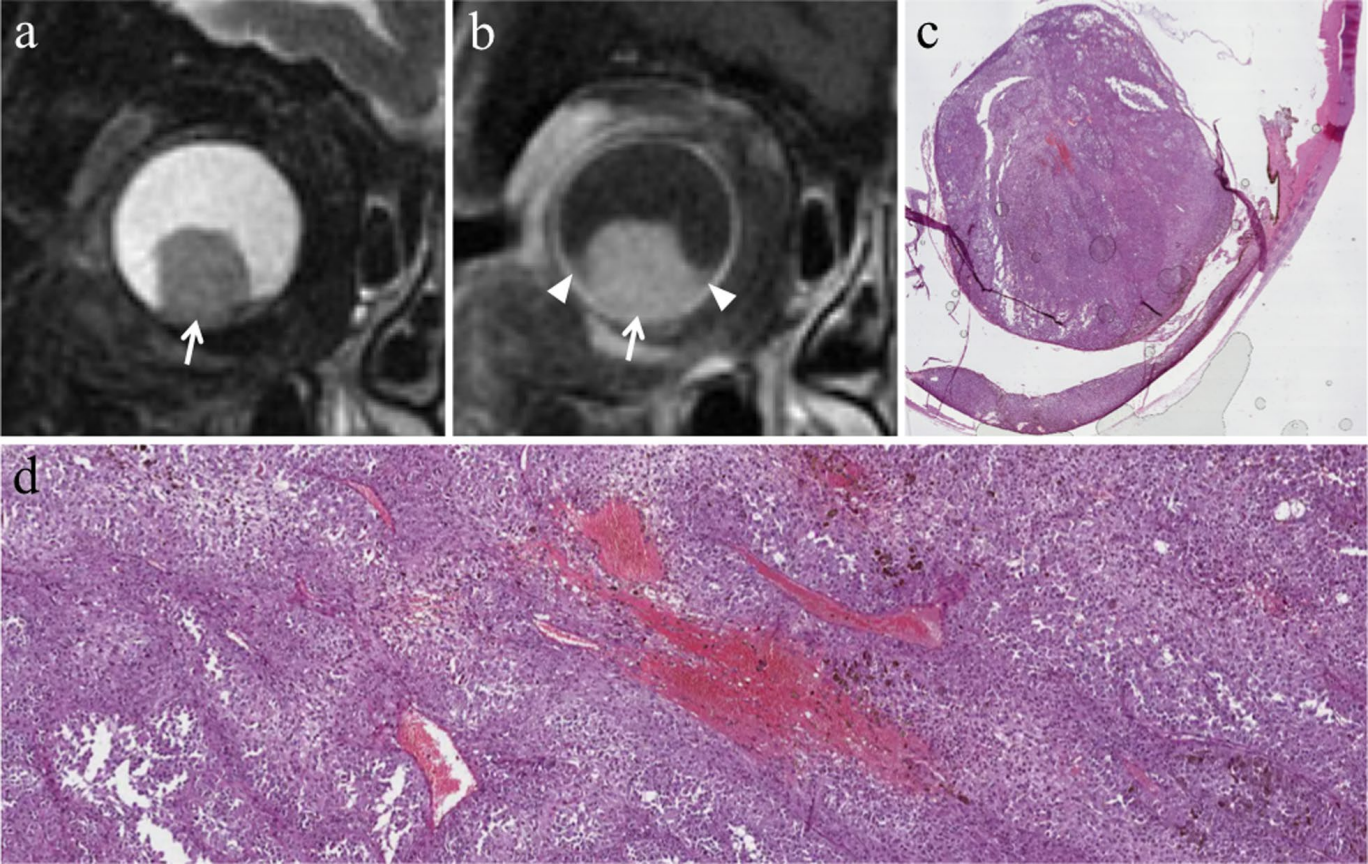
A 79-year-old woman with a poorly pigmented choroidal melanoma and hemorrhagic retinal detachment of the right eye. Coronal (**a**) T2-weighted turbo spin-echo STIR and (**b**) contrast-enhanced fat-suppressed T1-weighted images show a dome-shaped intraocular lesion along the inferior aspect of globe (white arrows), exhibiting intermediate signal intensity on T2-weighted image and enhancement on contrast-enhanced T1-weighted image. Note the small non-enhancing retinal detachment on both sides of the mass (white arrowheads in** b**). (**c**) Histological examination: the radiologically described mass on the posterior wall of the eye is well evident on low magnification; melanoma cells appear poorly pigmented (H&E, original magnification ×25). (**d**) Higher magnification demonstrating ectatic vessels, responsible for the enhancement of the lesion on post-contrast images. Note the lack of pigment and the presence of scattered melanophages intermingled with neoplastic cells (H&E, original magnification ×100)

**Fig. 9:**
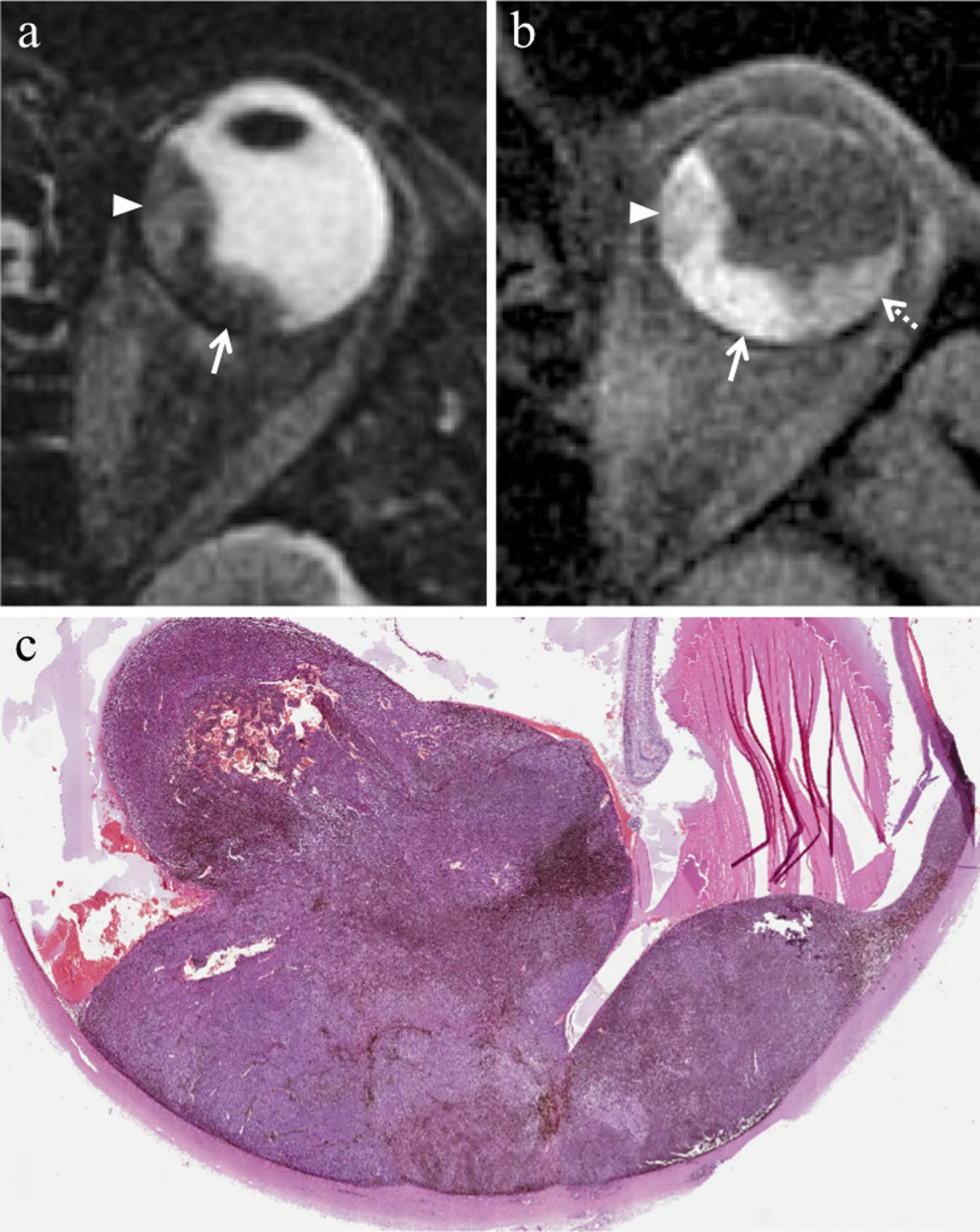
A 55-year-old man with a bipartite choroidal melanoma of the left eye. Axial (**a**) T2-weighted turbo spin-echo STIR and (**b**) fat-suppressed T1-weighted images show an intraocular bilobate mass with bipartite pigmentation along the postero-medial aspect of the globe. The medial portion of the lesion (white arrowheads) exhibits intermediate signal intensity on both T2- and T1-weighted images, whereas the posterior portion (white arrows) demonstrates predominantly low signal intensity on T2-weighted image and high signal intensity on T1-weighted image, due to melanin pigmentation. Laterally to the lesion a retinal detachment is better depicted on fat-suppressed T1-weighted image (white dotted arrow in** b**). (**c**) Histological examination: low magnification confirming the bilobate morphology of the melanoma, consisting of a less pigmented medial portion (on the left) and a more pigmented posterior component (on the right) (H&E, original magnification ×25)

Nevertheless, this typical imaging appearance occurs in about 70% of uveal melanomas [47]. Poorly pigmented and amelanotic tumors (about 20% of cases) may show intermediate signal intensity on both T1- and T2-weighted sequences (Fig. 8) [32, 48, 50, 51]. Moreover, the pigmentation of the tumor can be homogeneous or mixed, with areas showing various shades of signal intensities within the lesion at MR imaging. Accordingly, in melanomas with mixed pigmentation only the melanotic parts exhibit high signal intensity and low signal intensity on T1- and T2-weighted images, respectively [51]. The possibility of a bipartite lesion, characterized by two portions showing different signal intensities at MR examination, has been described in about 21% of uveal melanomas (Fig. 9) [52].

According to some authors [51] the degree of pigmentation may have a prognostic role; in particular, a more pronounced pigmentation would be associated with a less favorable prognosis, although this hypothesis is not unanimously shared. The assessment of tumor pigmentation can be qualitative (based on visual evaluation) or quantitative, the latter demonstrating a better correlation with histopathologic examination. Quantitative assessment is based upon the ratio between melanomatous signal intensity and vitreous body signal intensity on both T1- and T2-weighted images. In this specific task T1-weighted images perform better than T2-weighted ones, showing a higher correlation with histopathologic findings (86% and 26%, respectively) [51].

In the assessment of tumor pigmentation MR is more accurate than ophthalmoscopy; indeed, MR allows a complete evaluation of the melanin distribution within the whole tumor, whereas ophthalmoscopy is able to evaluate just the ventral portion of the lesion. In addition to paramagnetic effect of melanin, also the histopathologic features of melanomas play an important role in determining their MR appearance. Uveal melanomas are hypercellular lesions characterized by densely crammed cellular bundles and this also accounts for the moderately low signal intensity on T2-weighted images [23, 53].

Macroscopic cystic necrotic alterations within the tumor are seldomly encountered and are seen at MR imaging as areas of relative hypointensity and hyperintensity compared with the surrounding neoplastic tissue on T1- and T2-weighted images, respectively [54].

Uveal melanomas are commonly solid tumors, cavitation being described in less than 1% of cases [21]. Cavitary uveal melanomas have been reported at both US and MRI. This kind of tumor generally occurs in the ciliary body, but the choroid and, more rarely, iris may be involved as well [55, 56]. Cavitary melanomas are characterized by single (unilocular) or multiple (multilocular) cystlike cavities with typically thick walls. At histopathology the cavities may contain serous fluid, pigment-laden macrophages or erythrocytes, but do not represent areas of necrosis [55–57]. At MRI the cystic spaces are isointense with the vitreous body and therefore appear hypointense on T1-weighted images and hyperintense on T2-weighted images; the thick septa are isointense with the surrounding neoplastic tissue [56]. Cavitary melanomas should not be confused with benign uveal cavitary lesions, such as ciliary body cysts (the latter generally smaller than cavitary melanomas and bounded by thin walls) in order to avoid delayed adequate treatment [21, 55–57].

Uveal melanoma involves, in order of decreasing prevalence, the choroid (90%), the ciliary body (7%) and the iris (3%) [51, 58].

**Fig. 10:**
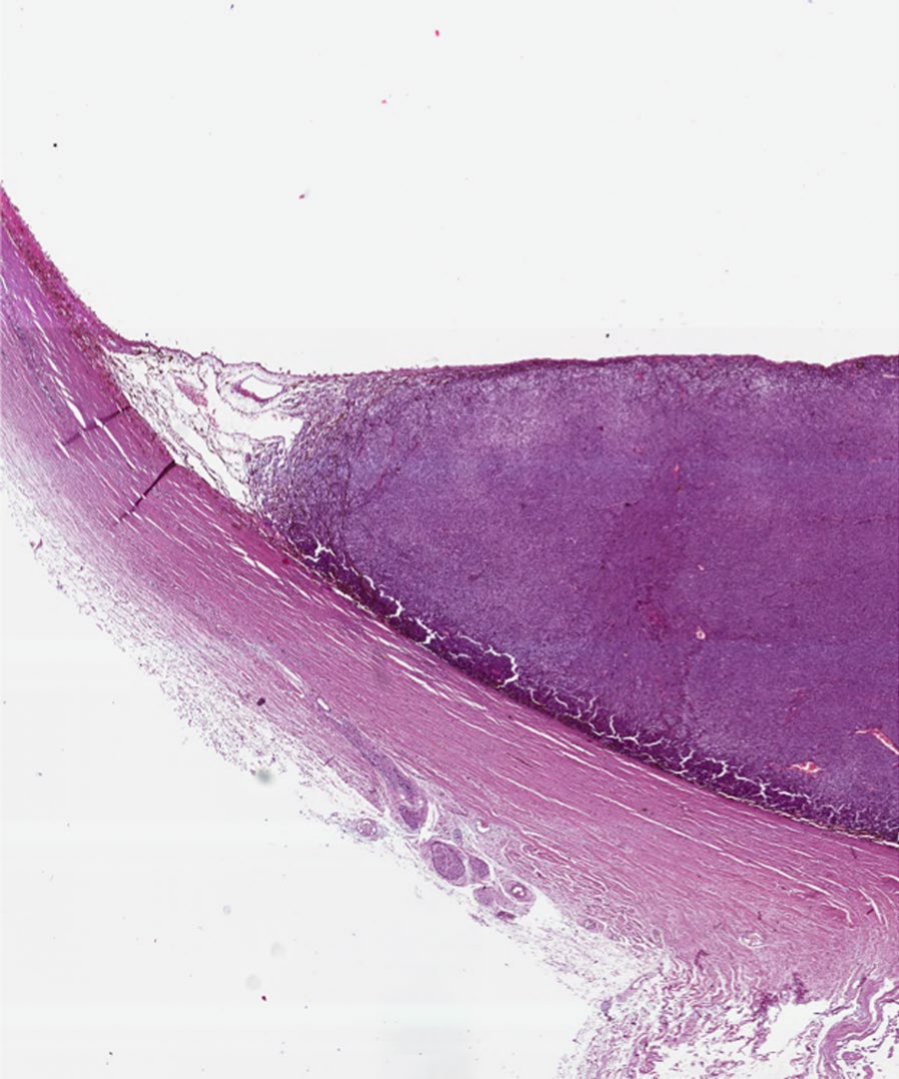
Histological examination demonstrating a melanoma originating from the choroid that raises up the retina (H&E, original magnification ×50)

**Fig. 11:**
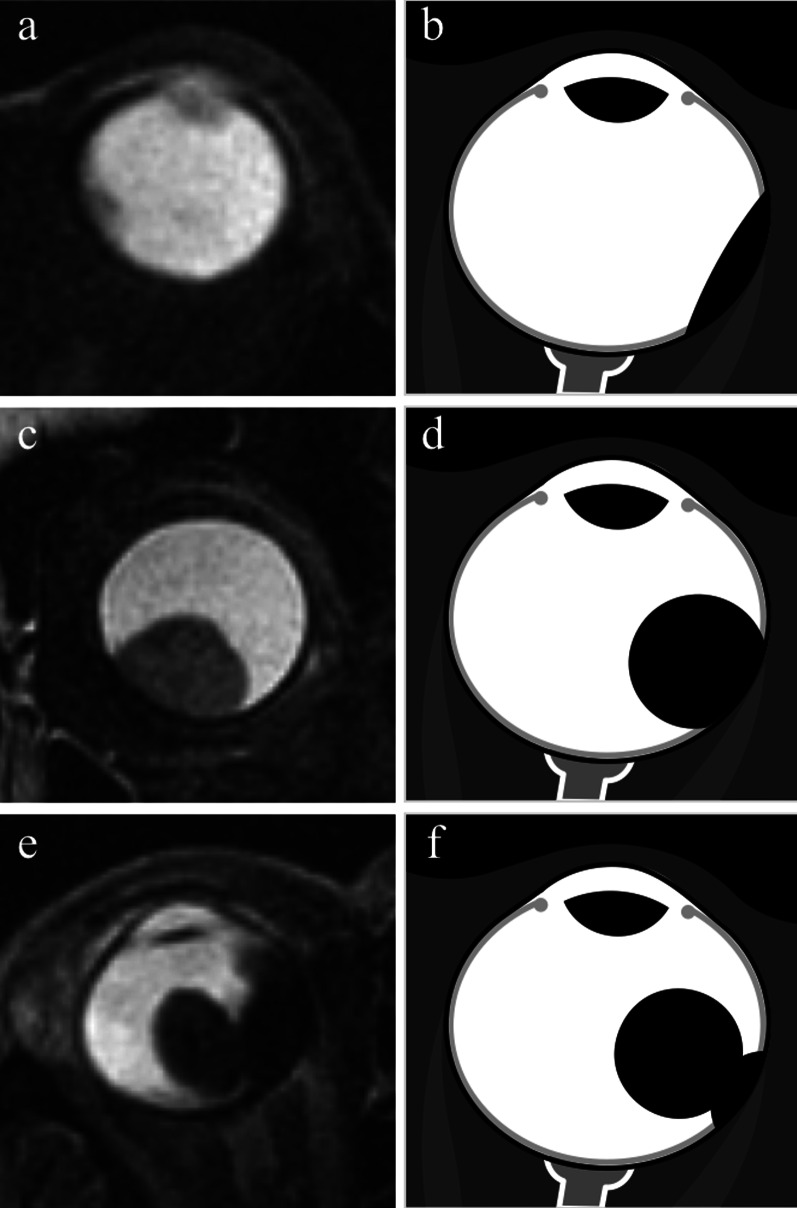
Different morphologic features of uveal melanomas. (**a, c, e**) Axial T2-weighted turbo spin-echo STIR images and (**b, d, f**) corresponding schematic drawings illustrate the three different macroscopic appearances of uveal melanomas: (**a, b**) lentiform shape or flat tumor, (**c, d**) mound or dome shape, (**e, f**) mushroom or collar-button shape

**Fig. 12:**
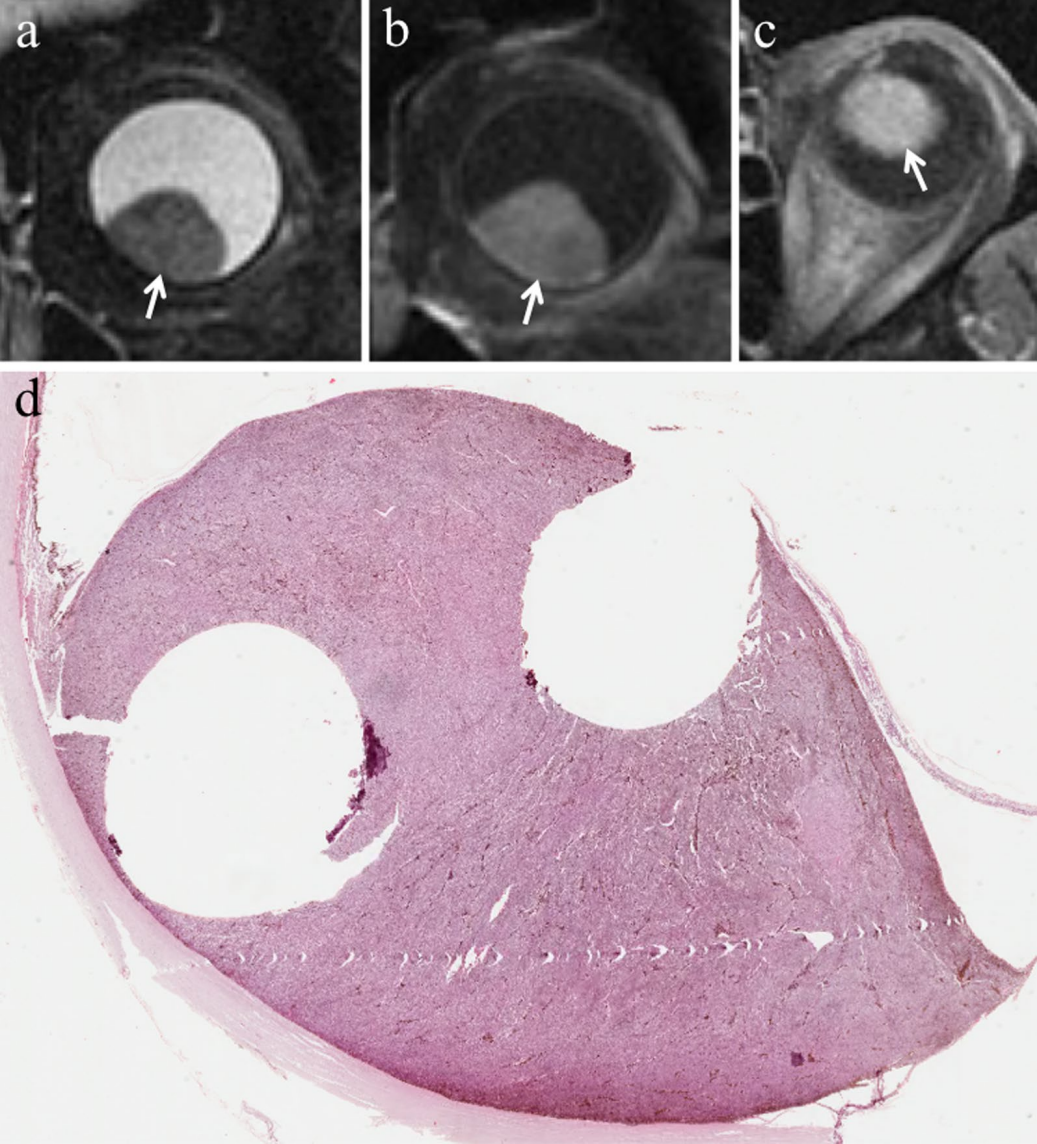
A 81-year-old woman with a dome-shaped choroidal melanoma of the left eye. (**a**) Coronal T2-weighted turbo spin-echo STIR image demonstrates an intraocular mass along the infero-medial aspect of the globe (white arrow). On (**b**) coronal and (**c**) axial contrast-enhanced fat-suppressed T1-weighted images the lesion displays enhancement (white arrows). (**d**) Histological examination displaying a protruding moderately pigmented mass with well circumscribed borders located in the posterior segment of the eye (H&E, original magnification ×25)

From a histological point of view, melanomas originate from the outer layer of the choroid and raise up the retina (Fig. 10). As a result of this, macroscopically, uveal melanomas can show three different morphologic features: lentiform shape or flat tumors (26.5%), mound or dome shape (37.5%), mushroom or collar-button shape (36%) (Fig. 11) [52]. Tumor shape has a prognostic relevance, inasmuch it mirrors the type of tumor development. Initially, lesions expanding along the choroid tend to assume a lentiform shape; however, when choroidal melanomas grow and break the Bruch’s membrane extending into the subretinal space, they acquire the typical mushroom shape, characterized by a stalk with a diameter smaller than that of the summit [26, 47, 48, 58]. Therefore, the mushroom shape indicates a progressive and infiltrative growth of the neoplasm. On the other hand, mound- or dome-shaped tumors demonstrate a displacing development (Fig. 12). Flat lesions may remain small over the years, but may also infiltrate the sclera demonstrating extraocular growth [47, 51].

Choroidal melanomas seldom (about 5% of cases) may produce diffuse infiltration of the choroid determining a homogeneous thickening (ring-shaped melanoma) associated with superficial retinal detachment [27, 53, 58].

**Fig. 13:**
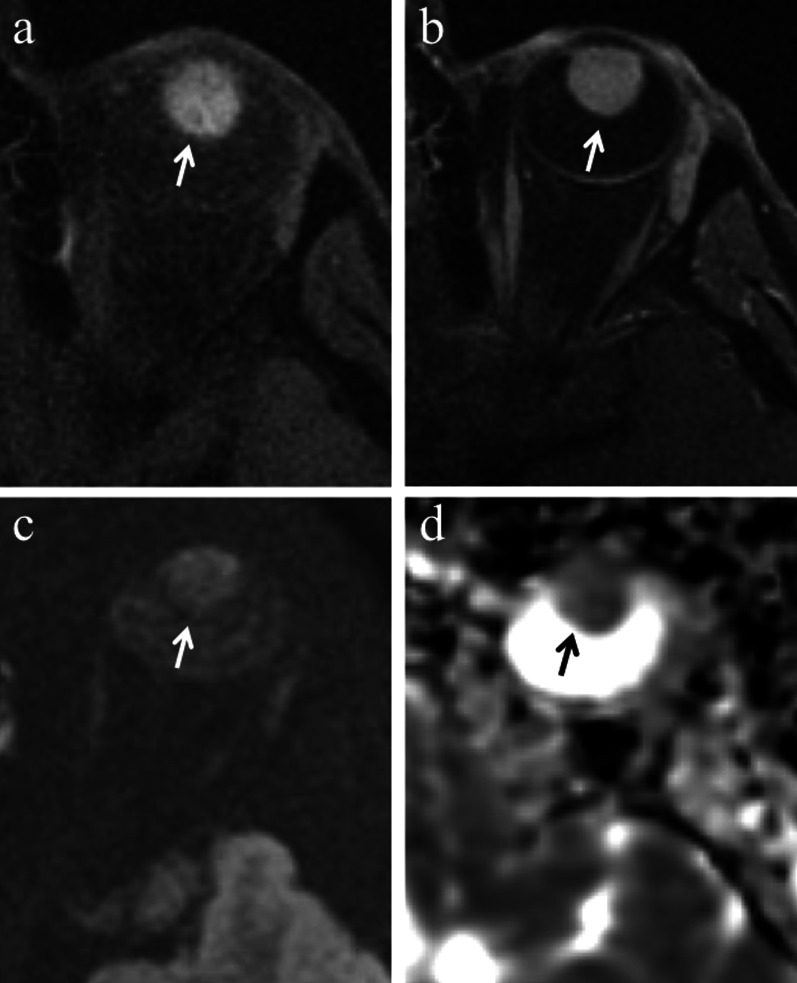
A 40-year-old man with a pigmented ciliary body melanoma of the left eye. The same patient as in Fig. 1. Axial (**a**) fat-suppressed T1-weighted and (**b**) contrast-enhanced fat-suppressed T1-weighted images illustrate an hyperintense mass along the anterior aspect of the globe (white arrows). After contrast agent administration, the lesion enhancement is hardly detectable due to the intrinsic hyperintensity of the lesion on pre-contrast T1-weighted image. On (**c**) axial DW image (b = 1000 s/mm^2^) and (**d**) corresponding ADC map the lesion exhibits restricted diffusion with high signal intensity (white arrow) on DWI image and low signal intensity (black arrow) on ADC map, a finding consistent with high cellularity

Uveal melanomas moderately enhance after intravenous administration of paramagnetic contrast agent, nevertheless lesion enhancement can be hardly detectable at simple visual evaluation due to the intrinsic T1 hyperintensity of the lesion (Fig. 13) [26].

Uveal melanoma is a malignant tumor, characterized by high cellularity, as mentioned before, and high nuclear/cytoplasmic ratio; accordingly, it shows noticeable restricted diffusion being hyperintense on DW images and hypointense on corresponding ADC map (Fig. 13c, d). The mean ADC value of ocular melanoma ranges from 891 × 10^–6^ to 1180 × 10^–6^ mm^2^/s in the various case series in the literature [37, 59, 60].

On DCE sequences uveal melanomas, like other malignant neoplasms, show a washout TIC pattern characterized by an initial increase followed by a subsequent decrease in signal intensity [26].

**Fig. 14:**
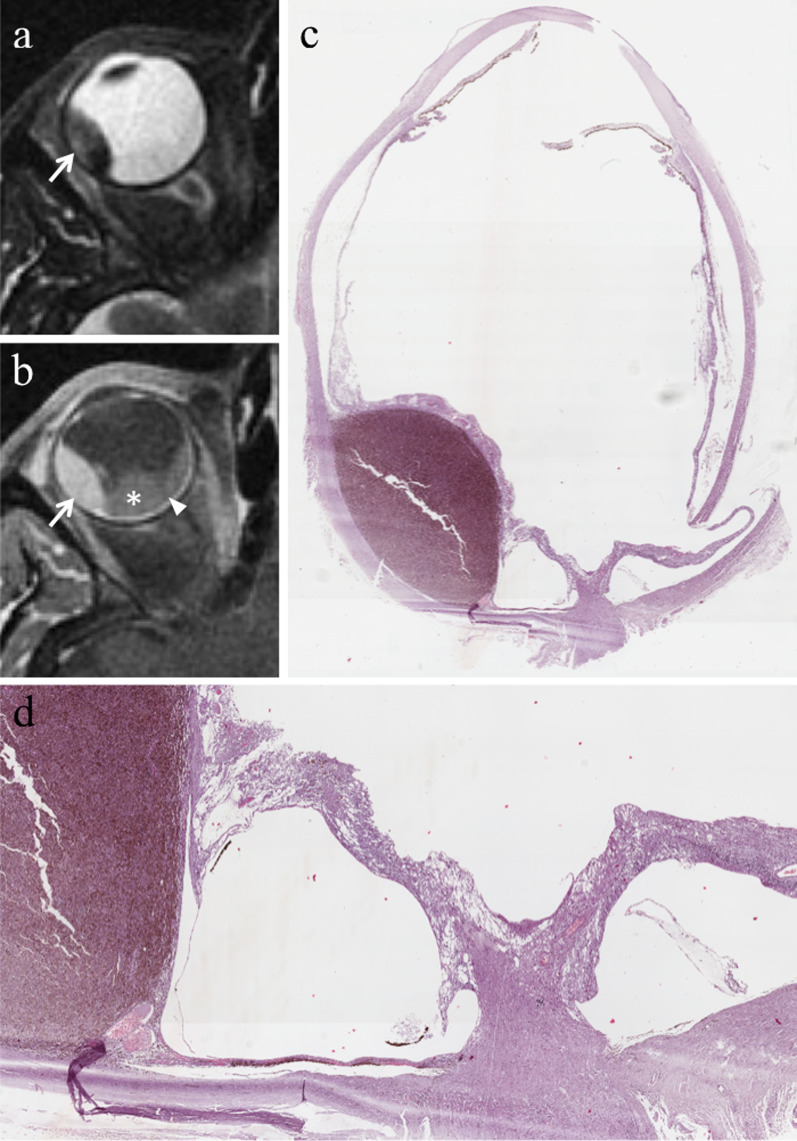
A 41-year-old woman with a pigmented choroidal melanoma and serous retinal detachment of the right eye. Axial (**a**) T2-weighted turbo spin-echo STIR and (**b**) contrast-enhanced fat-suppressed T1-weighted images show a pigmented choroidal melanoma along the lateral aspect of globe (white arrows); the lesion appears hypointense on T2-weighted image and hyperintense on contrast-enhanced fat-suppressed T1-weighted image. Along the posterior aspect of the globe a retinal detachment is hardly detectable on T2-weighted image, whereas it is better appreciable on fat-suppressed T1-weighted image (white asterisk in** b**) in which it does not enhance and demonstrates the typical biconvex V shape with the vertex in correspondence of the optic nerve head (white arrowhead). (**c, d**) Histology from a different patient from the one shown in MR images. The biconvex V-shaped retinal detachment, caused by a nearby intensely pigmented uveal melanoma, is readily identifiable on both (**c**) low (H&E, original magnifications ×25) and (**d**) high (H&E, original magnification ×50) magnification. Note the emergence of the optic nerve forming the vertex of the “V” (H&E, original magnifications ×25× and ×50, respectively)

In approximately 65% of patients, tumor growth determines retinal detachment, consisting in the split between the inner neurosensory retina and the underlying outer retinal pigment epithelium. Retinal detachment is deemed a manifestation of disease progression, although it is also related to individual factors. Retinal detachment has a typical imaging appearance showing a biconvex lentiform shape and a V shape with the vertex in correspondence of the optic nerve head and the extremities in the direction of the ora serrata (Fig. 14). This characteristic guise is due to anatomical reasons, as a consequence of retinal fastening to the flat portion of the ciliary body and to the optic nerve head [51]. When retinal detachment occurs, subretinal effusion can be: (a) exudative or serous, (b) hemorrhagic [47, 61], with signal intensity varying based on its protein content. Regardless of the kind of subretinal effusion, the retinal detachment is usually better demonstrated on T1-weighted sequences [23].

**Fig. 15:**
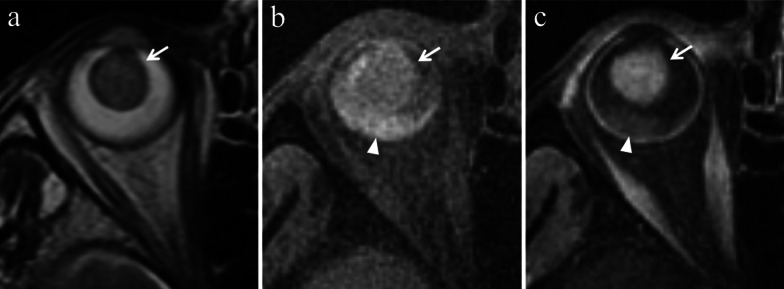
A 79-year-old woman with a poorly pigmented choroidal melanoma and hemorrhagic retinal detachment of the right eye. The same patient as in Fig. 8. Axial (**a**) T2-weighted turbo spin-echo STIR and (**b**) fat-suppressed T1-weighted images reveal an intraocular mass with intermediate signal intensity (white arrows), consistent with a poorly pigmented melanoma. Along the posterior aspect of the globe, the hemorrhagic retinal detachment is better depicted on fat-suppressed T1-weighted image in which it displays high signal intensity due to subacute blood products (white arrowhead). On (**c**) contrast-enhanced fat-suppressed T1-weighted image the lesion enhances (white arrow), whereas the retinal detachment does not enhance (white arrowhead)

**Fig. 16:**
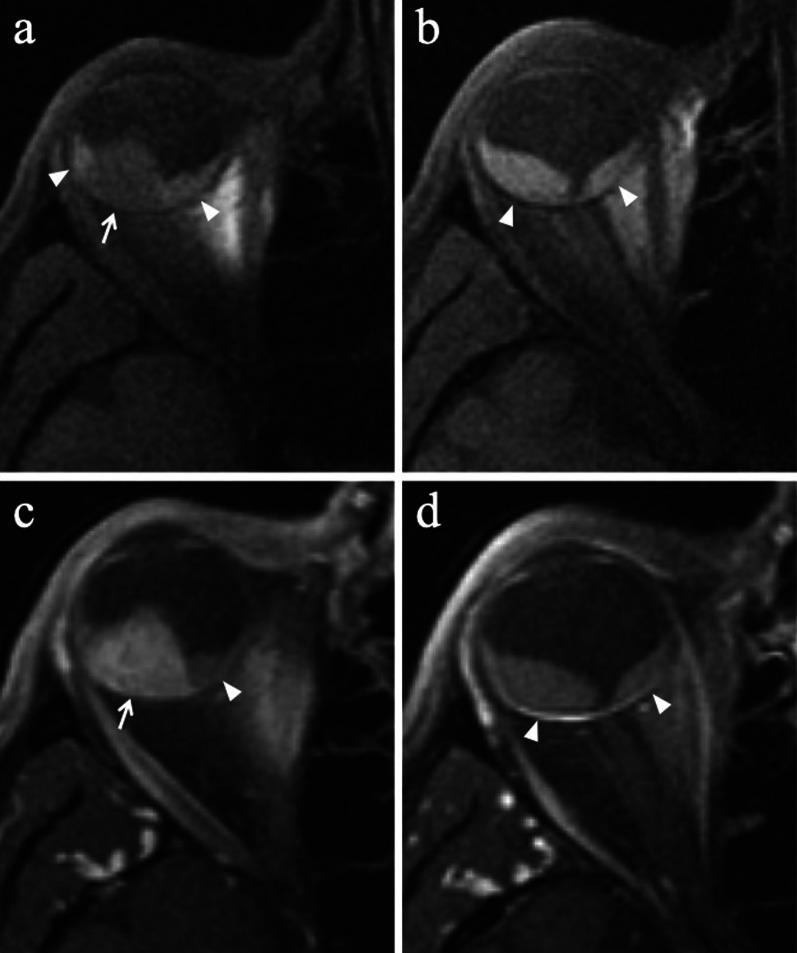
A 54-year-old man with a poorly pigmented choroidal melanoma and hemorrhagic retinal detachment of the right eye. (**a, b**) Axial fat-suppressed T1-weighted images demonstrate a dome-shaped intraocular mass along the postero-lateral aspect of globe (white arrow in** a**), displaying intermediate signal intensity because of low melanin content. A hemorrhagic retinal detachment, exhibiting the typical biconvex lentiform shape, is appreciable along the posterior aspect of globe (white arrowheads in b) and on both sides of the lesion (white arrowheads in** a**); the retinal detachment shows high signal intensity because of subacute hemorrhagic content. On (**c, d**) axial contrast-enhanced fat-suppressed T1-weighted images the choroidal melanoma exhibits vivid enhancement (white arrow in** c**), whereas the hemorrhagic retinal detachment does not enhance (white arrowheads)

To discriminate melanoma from retinal detachment is important in order to perform as reliable as possible tumor size measurements that have crucial implications in treatment planning and follow-up evaluation. Both Gd-based contrast agents and DWI are useful to distinguish retinal detachment from melanoma. In fact, uveal melanoma enhances and demonstrates restricted diffusion, whereas retinal detachment does not enhance and does not show restricted diffusion, unless hemorrhagic [26]. Only in this latter case retinal detachment may exhibit slight diffusion restriction and high signal intensity on T1-weighted sequences, because of subacute hemorrhagic content and paramagnetic effect of methemoglobin with T1 shortening (Figs. 7b, 15 and 16) [26]. For this reason, hemorrhagic retinal or choroidal detachment may also mimic melanoma at MRI [53]. The mean ADC of retinal detachment is 1986 × 10^–6^ mm^2^/s [59].

However, it is worth recalling that retinal detachment may also occur as a consequence of radiotherapy (secondary retinal detachment); in this case it tends to resolve in about three years [62, 63].

In around 7% of cases, uveal melanoma is associated with extraocular tumor growth [52]. In this regard it has to be remembered that MRI shows a sensitivity and a specificity of 100% and 50%, respectively, in the identification of scleral infiltration, and of 100% and 89% in the diagnosis of extrascleral extension [58].

## Differential diagnosis

Owing to its dual (mesodermal and neuroectodermal) origin, the uvea can represent the site of onset of tumors deriving from both mesoderm and neuroectoderm [53]. Differential diagnosis of uveal melanomas includes other uveal space-occupying lesions: choroidal nevus, choroidal hemangioma, melanocytoma, uveal metastases and choroidal detachment [32]. To distinguish uveal melanoma from other benign ocular lesions is of paramount importance in order to avoid unwarranted radiotherapy or even enucleation. Besides, it is important to remember that uveal melanoma can arise de novo or may originate from various worth mentioning preexisting predisposing lesions such as: uveal nevi, congenital melanosis, ocular and oculodermal melanocytosis [53, 64, 65].

### Choroidal nevus

Choroidal nevus, the most frequent benign intraocular neoplasm, represents a congenital lesion, generally located in the posterior third of the choroid; its reported prevalence ranges from 0.2% to 30% [65]. Choroidal nevi are commonly small (basal diameter ≤ 5 mm, height ≤ 1 mm), notwithstanding may be associated with superficial retinal detachment at times [53, 66].

Due to its melanin content, choroidal nevus may show a very similar MR imaging appearance to that of uveal melanoma, demonstrating high signal intensity on T1-weighted images and low signal intensity on T2-weighted images [54]. In these cases, long-term follow-up may be necessary to formulate a proper diagnosis [53]. In any case, choroidal nevus should be periodically evaluated for possible transformation into melanoma. In particular, the annual rate of malignant transformation of a choroidal nevus increases with age and is estimated at 1 in 8845 [65]. In this respect, it is important to remember that a small growth ≤ 1 mm in diameter over a period of 10 years usually represents a physiological expansion without implication for malignancy; on the other hand, a proved growth over a relatively limited period (< 5 years) is suspicious for malignant transformation [67].

### Choroidal hemangioma

Choroidal hemangioma (CH) is a congenital benign vascular hamartoma originating from the choroid. Two distinct clinic-pathologic entities of CH have been described: (a) the diffuse angiomatosis occurring as part of Sturge-Weber syndrome; (b) the sporadic, solitary or circumscribed form. In the former case the diagnosis is relatively easy, based upon other associated signs such as: facial nevus flammeus, homolateral cutaneous lesions, leptomeningeal hemangioma. On the other hand, the solitary CH is not related to Sturge-Weber syndrome and represents a major differential diagnosis of uveal melanoma [53, 68]. The circumscribed CH is usually entirely located posteriorly to the equator, mostly in the juxtapapillary region. The association with vitreous hemorrhage or retinal detachment can make it difficult or even impossible the clinical diagnosis of this tumor. Moreover, since CH is an amelanotic lesion, its differential diagnosis with amelanotic uveal melanoma can be challenging also under physiological conditions [23, 53, 68].

**Fig. 17:**
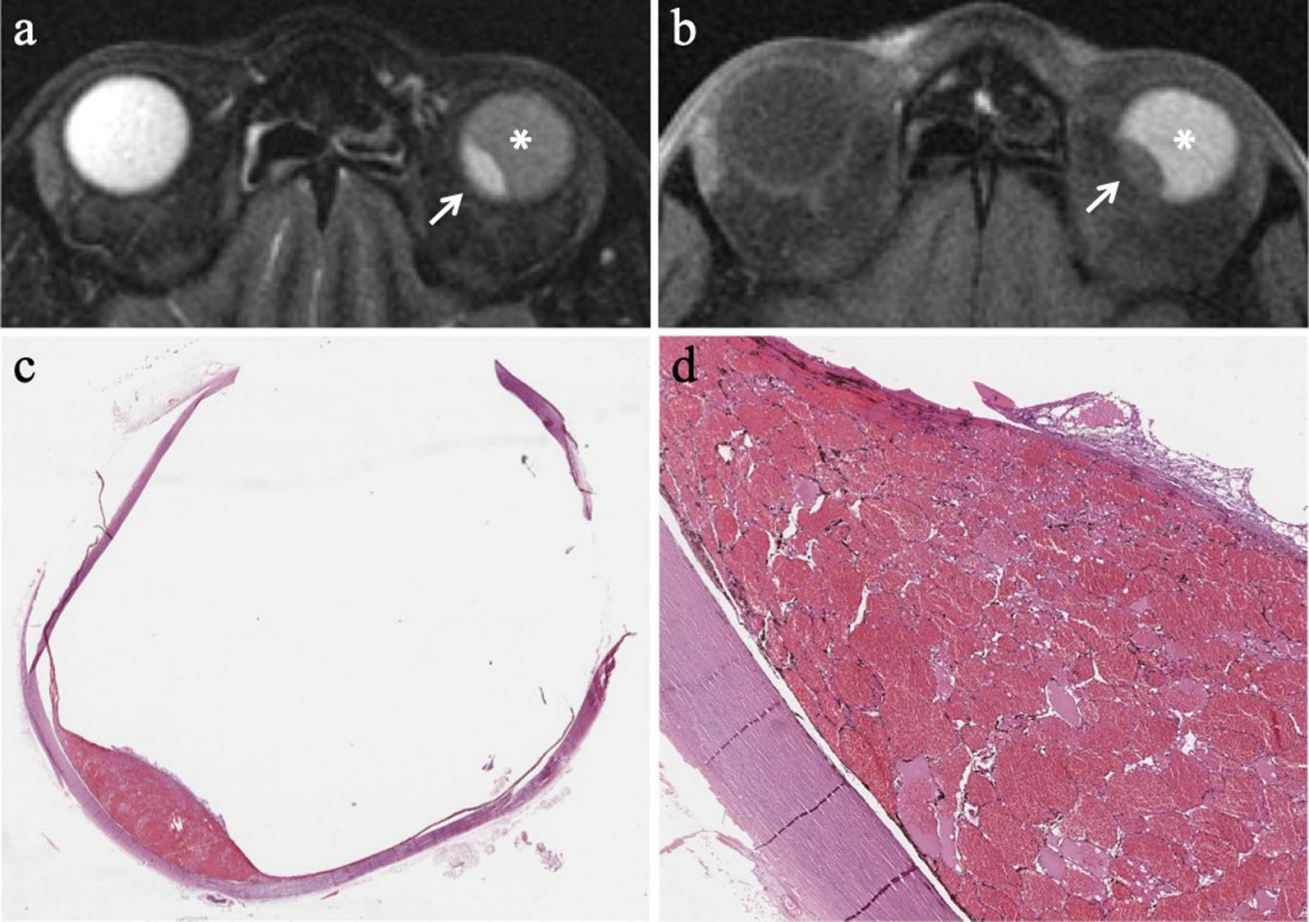
A 53-year-old woman with a choroidal hemangioma of the left eye treated with photodynamic therapy. The patient underwent secondary enucleation about seven years after the treatment because of vitreous hemorrhage. Axial (**a**) T2-weighted turbo spin-echo STIR and (**b**) fat-suppressed T1-weighted images show a lentiform intraocular mass along the postero-medial aspect of the globe (white arrows). Compared with the contralateral (normal) vitreous body, the lesion is isointense on T2-weighted image and slightly hyperintense on T1-weighted image. The vitreous body of the left eye is diffusely hypointense on T2-weighted and hyperintense on T1-weighted sequences because of extensive vitreous hemorrhage (asterisks). (**c**) Histological examination: low magnification showing a nodular, vascular mass protruding into the posterior chamber of the eye (H&E, original magnification ×25). (**d**) At higher magnification the mass was composed of numerous, dilated, cavernous vascular spaces lined by flattened endothelial cells and filled with red blood cells and fibrinoid material (H&E, original magnification ×100). The findings are consistent with choroidal hemangioma

At MRI choroidal hemangioma generally shows a lentiform or slightly dome-shaped appearance. On T1-weighted sequences CH is isointense or slightly hyperintense as compared with the vitreous; on T2-weighted sequences the lesion demonstrates a homogeneous isointensity with vitreous body, considered the most distinctive MR finding of CH (Fig. 17). After intravenous administration of Gd-based contrast agent, on dynamic T1-weighted images the lesion exhibits an early and marked enhancement due to copious tumor vessels. The enhancement of CH is generally earlier and more intense than that of uveal melanoma. Conversely from uveal melanomas, CH does not demonstrate extraocular growth and hardly determine rupture of Bruch’s membrane [23, 68].

### Melanocytoma

Melanocytomas are benign pigmented tumors that may occur near or on the optic disk or in the uvea [69]. Melanocytomas of the optic disk are hamartomas constituted by pigmented melanocytes arranged among the axons of the optic nerve head [70]. Although previously described as a static hamartomatous proliferation, tumor growth has been described (10–15% of cases) as well as malignant transformation into melanoma, the latter occurring in about 2% of cases [71]; hence, annual follow-up is needed. Melanocytomas may extend beyond the margin of the optic disk, thus involving the adjacent choroid or retina [70]. On the other hand, melanomas affecting primarily the optic nerve are exceedingly uncommon, whereas the prevalence of optic nerve invasion by choroidal melanoma ranges between 5 and 7% [70].

At MR melanocytomas may resemble uveal melanomas, being markedly hypointense on T2-weighted sequences [53]. However, melanocytomas seldom produces subretinal fluid or hemorrhage [70, 71].

### Intraocular metastases

Together with uveal melanomas, intraocular metastases represent the most common ocular malignant neoplasm in adults. The most prevalent primary tumors are breast cancer and bronchial carcinoma. Metastases are situated in the posterior portion of the eyeball in most cases (about 80%) [47], since neoplastic emboli enter into the eye through posterior ciliary arteries [23, 53]. Metastases may involve both eyes in about 30% of cases; on the other hand, bilateral melanomas are exceptionally rare [23, 53]. Retinal detachment is less frequent in metastases (25%) than in melanomas (65.5%); moreover, the size of metastases is generally smaller than that of melanomas [47]. Tumor shape is another important clue in the differential diagnosis. Metastasis usually have a flat shape (placoid and lentiform), placoid type being the most frequent (65.9%); this latter morphological appearance does not occur in melanomas in which the dome and mushroom shape are most common [47].

At MRI, metastases tend to demonstrate a more homogeneous signal intensity than melanomas, nevertheless the differentiation of these two entities on the basis of signal intensity is challenging because of the frequent overlap of imaging findings. In particular, amelanotic or poorly pigmented melanomas cannot be distinguished from metastases merely according to their signal intensity on T1- and T2-weighted images [47]. As for quantitative MR measurements, on T1-weighted sequences the tumor/vitreous body signal intensity ratio of uveal melanomas (2.13) is higher than that of metastases (1.8) [52].

### Choroidal detachment

Choroidal detachment is the consequence of a fluid collection into the virtual suprachoroidal space (cf. section anatomical notes and MR anatomy of the eye). It can be serous or hemorrhagic, localized or diffuse. Both serous and hemorrhagic choroidal detachment may be caused by intraocular surgery and ocular trauma, in addition serous choroidal detachment may represent a complication of inflammatory diseases (scleritis, uveitis, endophthalmitis). In particular, localized hemorrhagic choroidal detachment may be confused with a choroidal melanoma [23].

**Fig. 18:**
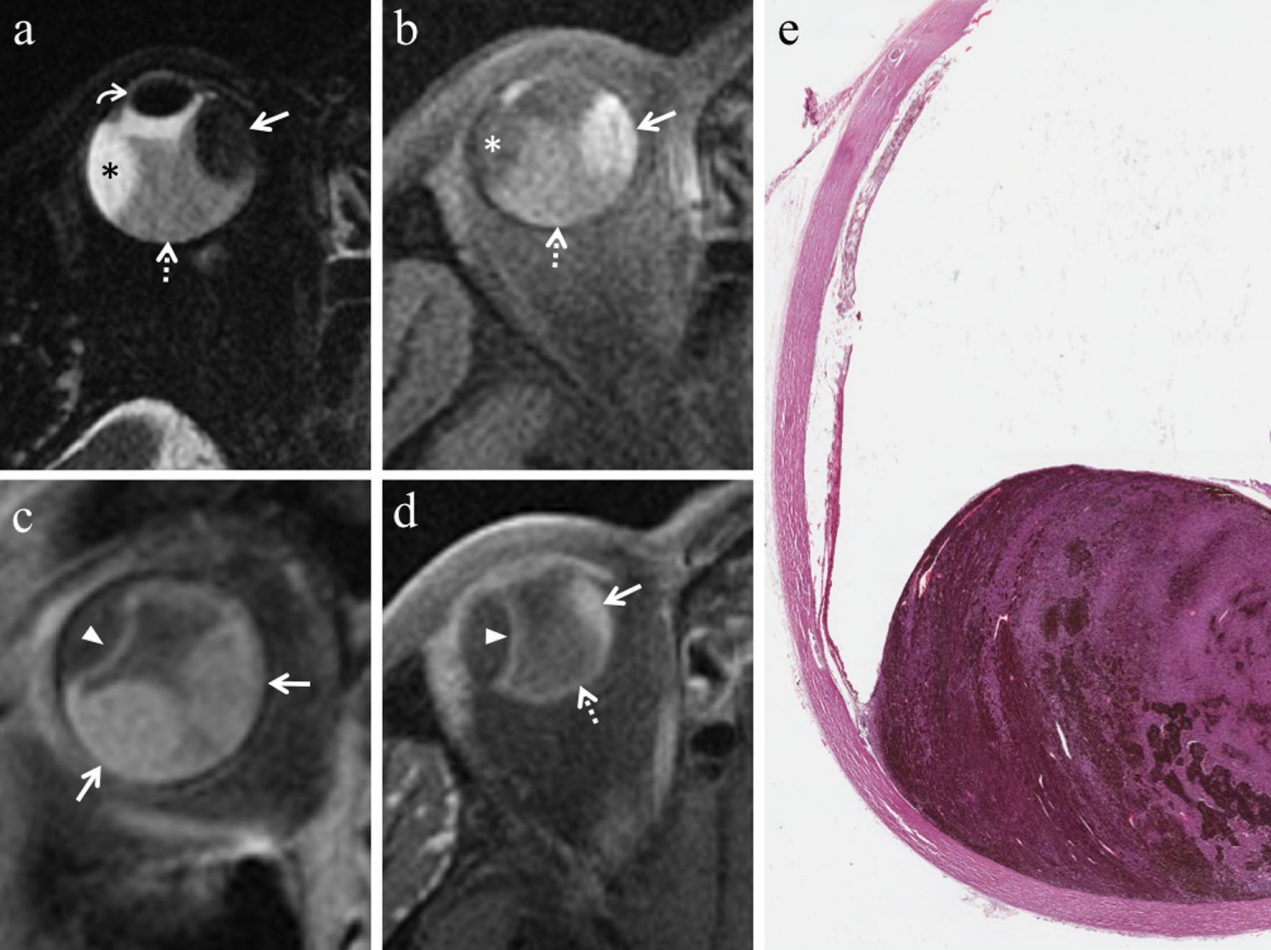
A 70-year-old man with serous choroidal detachment and hemorrhagic retinal detachment due to a large lobulated pigmented choroidal melanoma along the medial and inferior aspect of the right eye. Axial (**a**) T2-weighted turbo spin-echo STIR and (**b**) fat-suppressed T1-weighted images, (**c**) coronal and (**d**) axial contrast-enhanced fat-suppressed T1-weighted images. The intraocular mass along the medial and inferior aspect of the globe exhibits low signal intensity on T2-weighted image (white arrow in a), high signal intensity on T1-weighted image (white arrow in** b**) and mild enhancement after contrast agent administration (white arrows in** c** and** d**). Along the supero-lateral aspect of the eyeball, the localized serous choroidal detachment, with its well-defined lentiform shape, is hyperintense on T2-weighted (black asterisk in** a**) and hypointense on T1-weighted images (white asterisk in** b**); the detached choroid is easily appreciable after contrast agent administration (white arrowheads in** c** and** d**) because of its enhancement. The coexisting hemorrhagic retinal detachment demonstrates low signal intensity on T2-weighted image (white dotted arrow in** a**), high signal intensity on T1-weighted image (white dotted arrow in** b**) and does not enhance after contrast agent administration (white dotted arrow in** d**). The lens is displaced anteriorly (white curved arrow in** a**). **e** Histological examination: low magnification showing a pigmented uveal melanoma (below) and the coexisting choroidal detachment (on the left) (H&E, original magnification ×25)

At MRI, regardless of its content, a localized choroidal detachment appears as a focal, well defined, smooth, lentiform or, dome- or mound shaped mass. Serous choroidal detachment is hypointense on T1-weighted images and hyperintense on T2-weighted images (Fig. 18). On the other hand, the signal intensity of hemorrhagic choroidal detachment varies based on its age. In the first two days the hematoma appears slightly hypointense-isointense with the vitreous body on T1-weighted sequences and hypointense on T2-weighted sequences. Later, hemorrhagic choroidal detachment is hyperintense on T1-weighted and hypointense on T2-weighted images. Chronic hemorrhagic choroidal detachment (age ≥ 3 weeks) usually appears hyperintense on both T1- and T2-weighted sequences.

Retinal and choroidal detachments may have a quite similar appearance; however, some tips and tricks may allow to differentiate them. The leaves of the retinal detachment posteriorly stretch up to the site of the optic disk and anteriorly reach the ora serrata; on the other hand, the leafs of the choroidal detachment posteriorly do not reach the optic disk and anteriorly may involve the ciliary body, thus resulting in a ciliary detachment. Furthermore, the inner boundary of the choroidal detachment is constituted by the choroid and retina together, therefore enhances after i.v. administration of paramagnetic contrast agent and is thicker than that of the retinal detachment, represented by the retina alone (Table 4). Lastly, it should be remembered that, unlike retinal detachment which often accompanies uveal melanoma, choroidal detachment is very rarely associated with uveal melanoma. [23].

### Adenoma of the retinal pigment epithelium (RPE)

Adenoma of the retinal pigment epithelium (RPE) is a rare benign ocular neoplasm that may originate from congenital hypertrophy of the RPE. It is more often pigmented, but non-pigmented lesions have been reported as well [72–74]. Clinical manifestations are highly variable and nonspecific: floaters, blurred vision, eye fatigue [72, 73]. Usually it presents as a solid lesion with discrete margins in the posterior segment of the eye. On the basis of clinical examination, it can be challenging to differentiate the adenoma of the RPE from other pigmented tumors such as uveal melanoma or melanocytoma [72–74]. Nevertheless, some clinical and imaging features may suggest a correct diagnosis. Adenoma of the RPE is an abruptly elevated lesion, often associated with intraretinal and subretinal exudation, accompanied by a feeding retinal artery and a draining vein, serous retinal detachment may be present as well; the lesion size may be stationary or may show a very slow progression over time. On the other hand, uveal melanomas have a dome or mushroom shape, almost never demonstrate feeding and draining retinal vessels, and usually manifest a more rapid growth than RPE tumors [72–74].

At MRI adenoma of the RPE displays high signal intensity on T1-weighted images, low signal intensity on T2-weighted images and enhancement after administration of gadolinium-based contrast agents [72–74].

When diagnostic doubts persist, due to considerable overlap in some lesions, cytopathologic diagnosis through FNAB can be necessary [74]. Response to radiotherapy is poor [73]; on the other hand, endoresection performed by a skilled ophthalmologist represents a feasible surgical therapeutic option [72].

## Conclusion

Nowadays the radiologist provides a significant contribution to the clinical management of uveal melanoma. Of course, this pivotal position within a multidisciplinary team brings along with it various responsibilities too. Owing to its multiparametric and multiplanar capabilities, MR imaging has different advantages over the other imaging techniques, but equally requires a complete mastery of technical aspects and semeiotics. A detailed knowledge of the cross sectional anatomy along with the understanding of MR imaging appearance of uveal melanoma, both of its typical form and less frequent variants, are crucial for the characterization of the lesion and the differential diagnosis. Knowing how to deal with the technical issues that dramatically affect the quality of MR images is equally important. A careful assessment of the local extent of the disease is mandatory since it may considerably impact the choice of the therapeutic strategy. Lastly, the proper use of functional MR sequences (DWI) and quantitative biomarkers (ADC) both in the pretreatment assessment and in early follow-up may provide an important prognostic contribution. The continuous development of MR scanners and RF coil design coupled with the optimization of new MR sequences will further enhance the role of MRI in the diagnostic and therapeutic workup of uveal melanomas.

The following second instalment will present the therapeutic management of uveal melanoma.

## Data Availability

Data sharing is not applicable to this article as no new data were created or analyzed in this study.

## Abbreviations

ADC: Apparent diffusion coefficient
CH: Choroidal hemangioma
CT: Computed tomography
DCE-MRI: Dynamic contrast-enhanced magnetic resonance imaging
DWI: Diffusion weighted imaging
EPI: Echo planar imaging
FFA: Fundus fluorescein angiography
FNAB: Fine-needle aspiration biopsy
FOV: Field of view
MRI: Magnetic resonance imaging
OCT: Optical coherence tomography
PET/CT: Positron emission tomography/computed tomography
ROI: Region of interest
RPE: Retinal pigment epithelium
SAR: Specific absorption rate
SNR: Signal-to-noise ratio
STIR: Short inversion time inversion recovery
SUV: Standardized uptake value
TSE: Turbo spin-echo
UBM: Ultrasound biomicroscopy
US: Ultrasonography
